# C-type lectin receptor DCIR contributes to hippocampal injury in acute neurotropic virus infection

**DOI:** 10.1038/s41598-021-03201-2

**Published:** 2021-12-10

**Authors:** Melanie Stoff, Tim Ebbecke, Malgorzata Ciurkiewicz, Suvarin Pavasutthipaisit, Sabine Mayer-Lambertz, Theresa Störk, Kevin D. Pavelko, Wolfgang Baumgärtner, Klaus Jung, Bernd Lepenies, Andreas Beineke

**Affiliations:** 1 Department of Pathology, University of Veterinary Medicine Hannover, 30559 Hannover, Germany; 2 Center for Systems Neuroscience, 30559 Hannover, Germany; 3 Institute for Immunology and Research Center for Emerging Infections and Zoonoses, University of Veterinary Medicine Hannover, 30559 Hannover, Germany; 4 Department of Pathology, Faculty of Veterinary Medicine, Mahanakorn University of Technology, Bangkok, 10530 Thailand; 5 Department of Immunology, Mayo Clinic, Rochester, MN 55905 USA; 6 Institute for Animal Breeding and Genetics, University of Veterinary Medicine Hannover, 30559 Hannover, Germany

**Keywords:** Immunology, Antigen processing and presentation, Innate immunity, Neuroimmunology, Neuroscience, Neuroimmunology, Experimental models of disease

## Abstract

Neurotropic viruses target the brain and contribute to neurologic diseases. C-type lectin receptors (CLRs) are pattern recognition receptors that recognize carbohydrate structures on endogenous molecules and pathogens. The myeloid CLR dendritic cell immunoreceptor (DCIR) is expressed by antigen presenting cells and mediates inhibitory intracellular signalling. To investigate the effect of DCIR on neurotropic virus infection, mice were infected experimentally with Theiler’s murine encephalomyelitis virus (TMEV). Brain tissue of TMEV-infected C57BL/6 mice and DCIR^−/−^ mice were analysed by histology, immunohistochemistry and RT-qPCR, and spleen tissue by flow cytometry. To determine the impact of DCIR deficiency on T cell responses upon TMEV infection in vitro*,* antigen presentation assays were utilised. Genetic DCIR ablation in C57BL/6 mice was associated with an ameliorated hippocampal integrity together with reduced cerebral cytokine responses and reduced TMEV loads in the brain. Additionally, absence of DCIR favoured increased peripheral cytotoxic CD8^+^ T cell responses following TMEV infection. Co-culture experiments revealed that DCIR deficiency enhances the activation of antigen-specific CD8^+^ T cells by virus-exposed dendritic cells (DCs), indicated by increased release of interleukin-2 and interferon-γ. Results suggest that DCIR deficiency has a supportive influence on antiviral immune mechanisms, facilitating virus control in the brain and ameliorates neuropathology during acute neurotropic virus infection.

## Introduction

Neurotropic viruses target the brain and can cause asymptomatic or acute and fatal diseases^1,2^. Moreover, cognitive deficits and memory impairment, suggestive of hippocampal dysfunction, as well as an increased risk of developing epilepsy are often observed in patients surviving acute viral encephalitis^3–5^.

Theiler’s murine encephalomyelitis virus (TMEV) is a neurotropic picornavirus that preferentially infects the hippocampus^6–8^. While TMEV persists in the central nervous system (CNS) of SJL mice, C57BL/6 mice eliminate the virus following acute polioencephalitis. C57BL/6 mice mount early robust antiviral immune responses, but are prone to develop hippocampal injury with neuronal loss during the acute infection phase^1,9^. TMEV infection was shown to increase the susceptibility to develop seizures in C57BL/6 mice^6,7^. Moreover, neuronal damage is associated with impaired cognition and spatial memory, making TMEV infection a valuable model for brain damage in neurotropic virus infections^10–12^. Innate immune responses during the initial phase significantly contribute to the development of antiviral T cell responses and virus elimination in TMEV-infected mice^13–15^. However, CNS-infiltrating macrophages and activated microglia also account for hippocampal degeneration following TMEV infection by releasing pro-inflammatory factors^16–21^. Surveillance of virus infection and initiation of innate immune responses are mediated by pattern recognition receptors (PRRs) on professional antigen presenting cells (APCs), such as dendritic cells (DCs). C-type lectin receptors (CLRs) are PRRs that recognise a variety of glycan structures present on pathogens, including viruses, damage-associated molecular patterns and self-glycoproteins^22–27^.

The C-type lectin receptor Dendritic cell immunoreceptor (DCIR, human gene: CLEC4A, murine gene: Clec4a2) contains an immunoreceptor tyrosine-based inhibition motif (ITIM), which delivers inhibitory signals predominantly in DCs^28–31^. Thus, DCIR is a negative regulator of intracellular signalling of APCs, including microglia, and contributes to immune homeostasis in immune mediated disorders^31–34^. However, DCIR signalling seems to play an ambiguous role in infectious diseases^30,35,36^. For instance, while DCIR signalling limits immunopathology in Chikungunya virus-infected mice, the receptor is thought to trigger brain pathology in cerebral malaria models^37,38^. The role of DCIR signalling in neurotropic virus infections and its impact on neuropathology have not yet been investigated.

The aim of the present study was to investigate DCIR-mediated effects on the balance of immune responses, virus load and neuropathology in Theiler’s murine encephalomyelitis (TME). Genetic ablation of DCIR indicates that receptor signalling contributes to neuroinflammation and brain injury in C57BL/6 mice following TMEV infection. DCIR^−/−^ mice show an improved hippocampal integrity and are able to control neurotropic virus infection more efficiently. In vitro studies reveal that DCIR deficiency enhanced T cell activation in a dendritic cell/T cell co-culture system.

## Results

### DCIR^−/−^ mice show preservation of hippocampal integrity and reduced viral load in the brain

**Figure 1:**
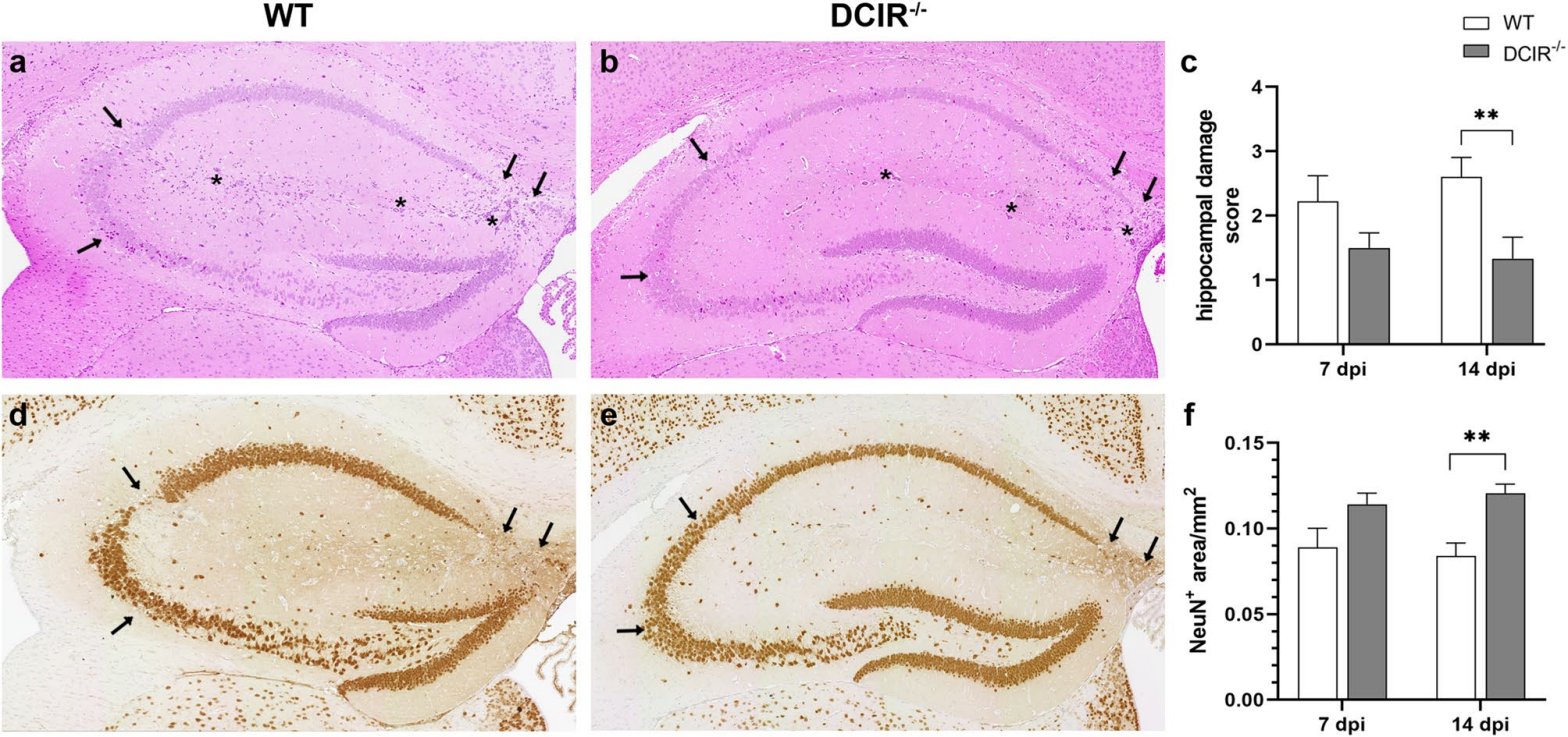
Neuropathology and neuronal loss within the hippocampus following Theiler’s murine encephalomyelitis virus (TMEV) infection. (**a**–**c**) Diminished hippocampal damage in DCIR^−/−^ mice. (**a**, **b**) Representative images from 14 dpi show neuronal loss (arrows) and perivascular inflammatory infiltrates (asterisk) in the hippocampus of wild type (WT) and DCIR^−/−^ mice (hematoxylin and eosin staining). (**d**–**f**) Decreased neuronal loss in DCIR^−/−^ mice confirmed by NeuN-specific immunohistochemistry. (**d**, **e**) Representative images from 14 dpi show neuronal loss of hippocampal pyramidal layer (arrows) in WT and DCIR^−/−^ mice. (**c**, **f**) Statistical analysis: Mann–Whitney *U* test (statistical differences: *p ≤ 0.05, **p ≤ 0.01); data are shown as mean with SEM. n: 7 dpi = 9 WT and 12 DCIR^−/−^ mice; 14 dpi = 10 WT and 9 DCIR^−/−^ mice. (**a**, **b**, **d**, **e**) 200 × magnification; *dpi * days post infection.

The effect of DCIR deficiency on hippocampal and neuronal integrity following TMEV infection was determined (Fig. 1). Two-way ANOVA yielded a significant effect of DCIR deficiency on neuronal integrity determined by histology (HE score; *p* = 0.0008) and immunohistochemistry (NeuN^+^ area/mm^2^, *p* = 0.0006), as well as on TMEV load (TMEV^+^ cells/mm^2^, *p* = 0.0257). Subsequent Mann–Whitney *U* tests at different time points post infection revealed a diminished hippocampal damage of infected DCIR^−/−^ mice with a significant difference compared to WT mice at 14 dpi (*p* = 0.005, Fig. 1a–c). Similarly, a significantly reduced loss of NeuN^+^ neurons in the hippocampus of DCIR^−/−^ animals compared to WT controls was found at 14 dpi (*p* = 0.002, Fig. 1d–f). Although increased numbers of β-APP^+^ axons (damaged axons) were found in hippocampal regions with severe neuronal damage and loss mainly in TMEV-infected WT mice, group differences did not reach the level of significance (Supplementary Fig. S1a). Likewise, an elevation of GFAP^+^ astrocytes (astrogliosis) was present within the hippocampus of WT animals compared to DCIR^−/−^ mice at 14 dpi, but differences did not reach the level of significance (Supplementary Fig. S1b). No hippocampal inflammation and damage were found in non-infected, age-matched WT and DCIR^−/−^ animals. In addition, non-infected groups showed a similar amount of NeuN^+^ neurons and GFAP^+^ astrocytes in the hippocampus (Supplementary Fig. S5).

**Figure 2:**
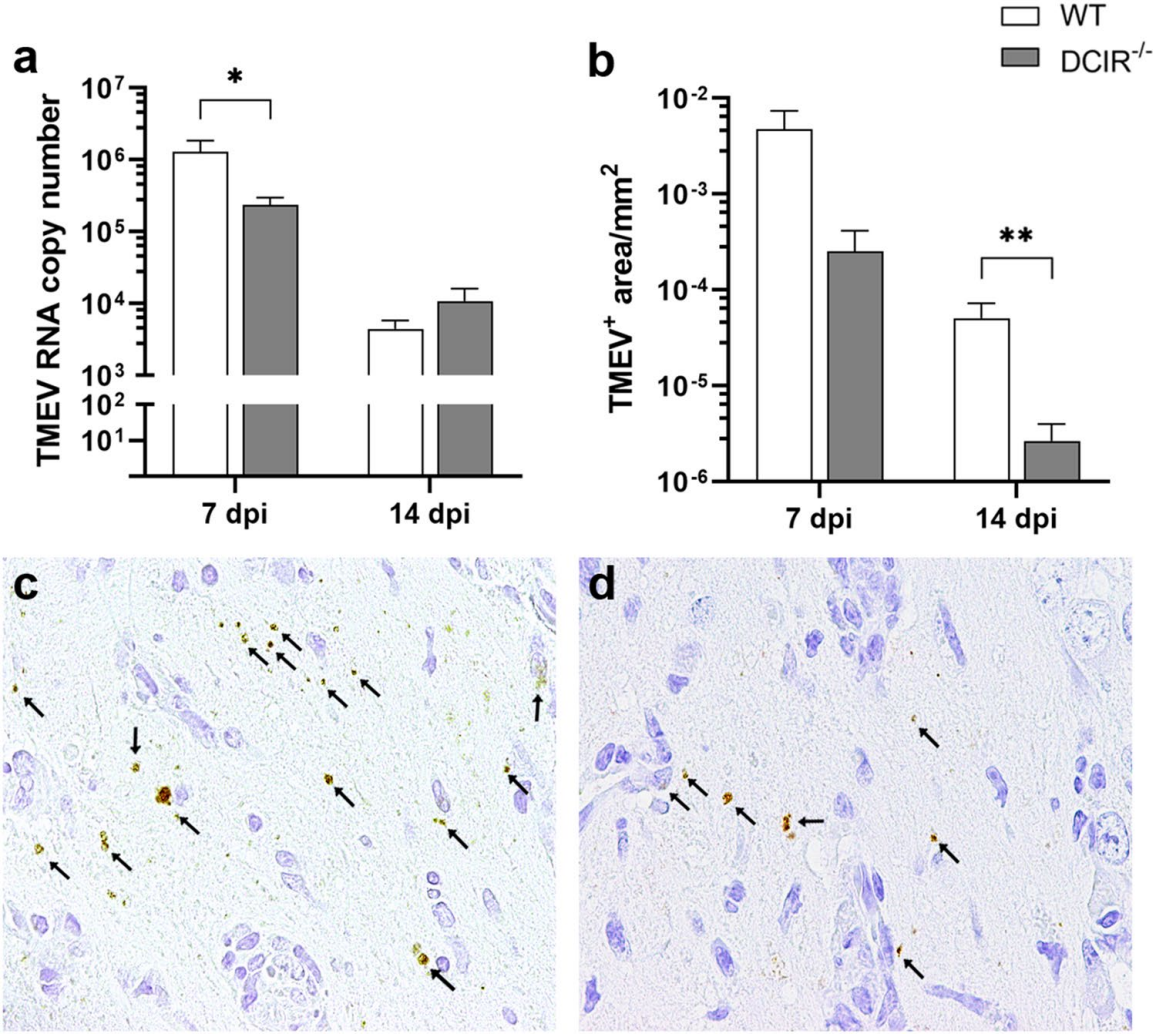
Theiler’s murine encephalomyelitis virus (TMEV) infection in the brain. (**a**) Quantification of TMEV RNA concentrations by reverse transcriptase quantitative polymerase chain reaction. (**b–d**) Quantification of the TMEV^+^ area within the hippocampus by immunohistochemistry. Representative images show TMEV antigen (arrows) in the hippocampus of (**c**) wild type (WT) and (**d**) DCIR^−/−^ mice at 14 dpi. (**a**, **b**) Statistical analysis: Mann–Whitney *U* test (statistical differences: *p ≤ 0.05, **p ≤ 0.01); data are shown as mean with SEM. n: 7 dpi = 9 WT and 12 DCIR^−/−^ mice; 14 dpi = 10 WT and 9 DCIR^−/−^ mice. (**c**, **d**) 600 × magnification; *dpi * days post infection.

Viral quantification within the brain was performed by RT-qPCR and TMEV-specific immunohistochemistry. At 7 dpi, TMEV RNA concentration was significantly decreased in DCIR^−/−^ mice compared to WT mice (*p* = 0.047, Fig. 2a). Immunohistochemistry revealed a preferential infection of hippocampal neurons of infected mice in both groups at 7 dpi. Similar to the diminished viral RNA load, reduced numbers of TMEV-infected cells were observed in the brain of DCIR^−/−^ mice at 7 dpi, but differences did not reach level of significance (*p* = 0.12, Fig. 2b). Both, WT and DCIR^−/−^ mice, showed reduced viral RNA levels and TMEV antigen at 14 dpi, indicating viral elimination (Fig. 2a,b). TMEV RNA concentration in the brain did not differ significantly between both groups at 14 dpi (*p* = 0.87, Fig. 2b). However, the number of TMEV^+^ cells within the hippocampus was significantly reduced at 14 dpi in DCIR^−/−^ mice compared to WT mice, indicating a reduced residual infection following acute infection phase in DCIR deficient animals (*p* = 0.005, Fig. 2b–d). No TMEV was detected in non-infected WT mice and DCIR^−/−^ mice by immunohistochemistry and RT-qPCR (data not shown). Data show that preserved hippocampal morphology in mice lacking DCIR is associated with an enhanced early virus elimination from the brain, indicating a refined induction of protective responses in DCIR^−/−^ mice following TMEV infection.

Weekly clinical examination, body weight recordings, Racine score evaluation and RotaRod performance test revealed no symptoms in both groups, indicating a subclinical acute infection (Supplementary Fig. S4)^39^.

### DCIR deficiency leads to diminished brain sequestration of effector immune cells

**Figure 3:**
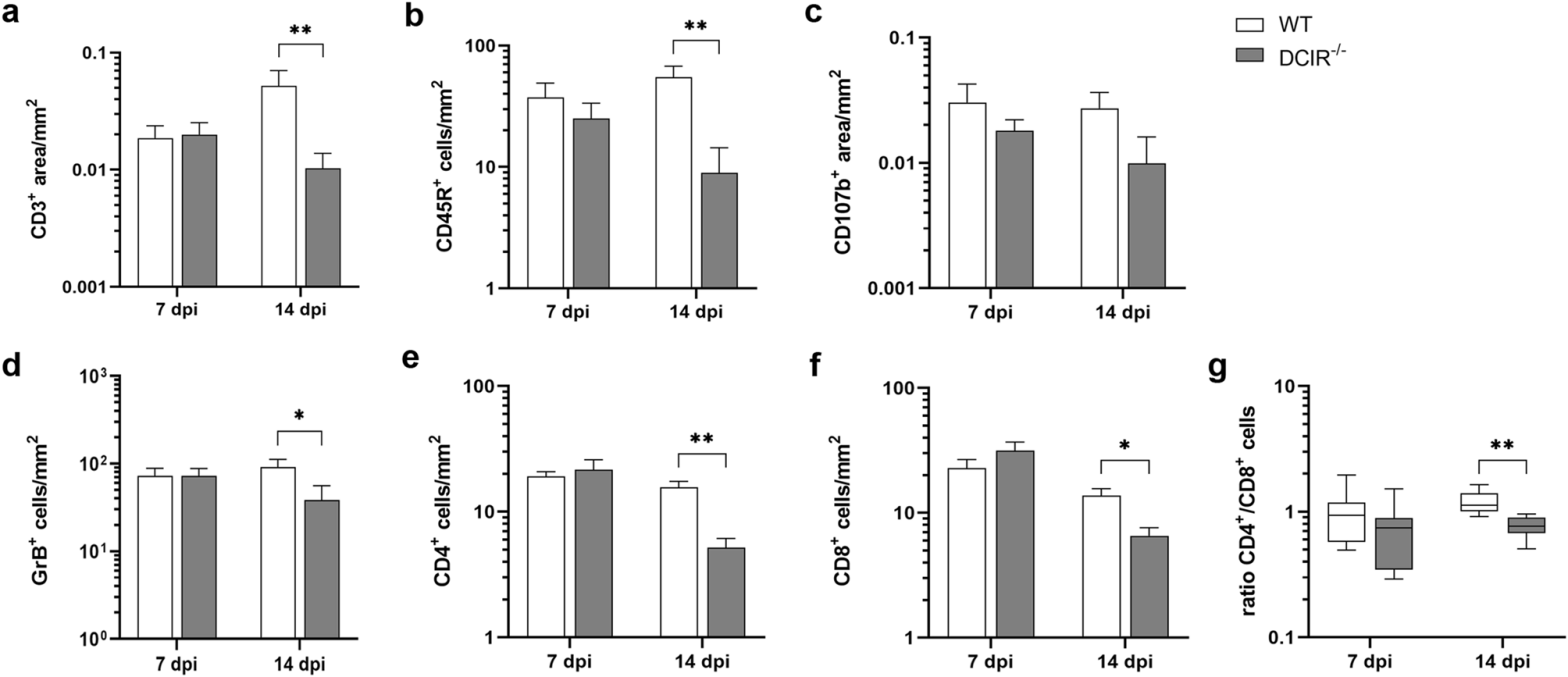
Phenotyping of lymphocyte subsets in the hippocampus following Theiler’s murine encephalomyelitis virus (TMEV) infection. Quantification of (**a**) CD3^+^ T cells, (**b**) CD45R^+^ B cells, (**c**) CD107b^+^ macrophages/microglia, (**d**) GrB^+^ cells, (**e**) CD4^+^ T cells, and (**f**) CD8^+^ T cells in the hippocampus of infected wild type (WT) and DCIR^−/−^ mice, and (**g**) ratio of CD4^+^ T cells to CD8^+^ T cells in the cerebrum determined by immunohistochemistry. (**a**–**g**) Statistical analysis: Mann–Whitney *U* test (statistical differences: *p ≤ 0.05, **p ≤ 0.01); data are shown as mean with SEM (**a**–**f**) and box plots display median with 5–95% percentiles (**g**). n: 7 dpi = 9 WT and 12 DCIR^−/−^ mice; 14 dpi = 10 WT and 9 DCIR^−/−^ mice. *dpi * days post infection.

Immunohistochemistry revealed a reduced infiltration of CD3^+^ T cells (*p* = 0.007, Fig. 3a) and CD45R^+^ B cells (*p* = 0.005, Fig. 3b) in the hippocampus of DCIR^−/−^ mice compared to WT controls at 14 dpi. At both time points, the hippocampus of DCIR^−/−^ mice contained similar numbers of activated CD107b^+^ macrophages/microglia in comparison to WT controls (Fig. 3c).

Brain-infiltrating GrB^+^ effector cells decreased in DCIR^−/−^ mice at 14 dpi (*p* = 0.05, Fig. 3d). Moreover, reduced numbers of CD4^+^ and CD8^+^ T cells were found in the hippocampus of DCIR^−/−^ mice at 14 dpi (CD4^+^ T cells: *p* = 0.002, Fig. 3e, CD8^+^ T cells: *p* = 0.011, Fig. 3f), likely related to decreased virus-trigged immune responses in receptor deficient animals. Comparison of CD4^+^ and CD8^+^ T cell proportions revealed a slightly reduced ratio of CD4^+^ to CD8^+^ T cells in the brain of DCIR^−/−^mice at 14 dpi (*p* = 0.00045, Fig. 3g), showing a relative increase of cytotoxic T cells in animals lacking DCIR. Statistical analyses (Pearson’s correlation coefficient R) revealed negative correlations between neuronal integrity of the hippocampus (NeuN^+^ area/mm^2^) and the amount of CD107b^+^, arginase 1^+^ and CD45R^+^ cells at 7 and 14 dpi. Foxp3^+^ cells and GFAP^+^ astrocytes were negatively correlated with neuronal integrity at 14 dpi (Supplemental Table S6).

Non-infected control animals of both groups showed no leukocyte infiltrations in the hippocampus.

Collectively, these findings suggest that the reduced viral brain load in DCIR^−/−^ mice leads to an accelerated termination of brain inflammatory responses.

### Reduced cerebral cytokine expression in DCIR^−/−^ mice is associated with preserved hippocampal integrity

**Figure 4:**
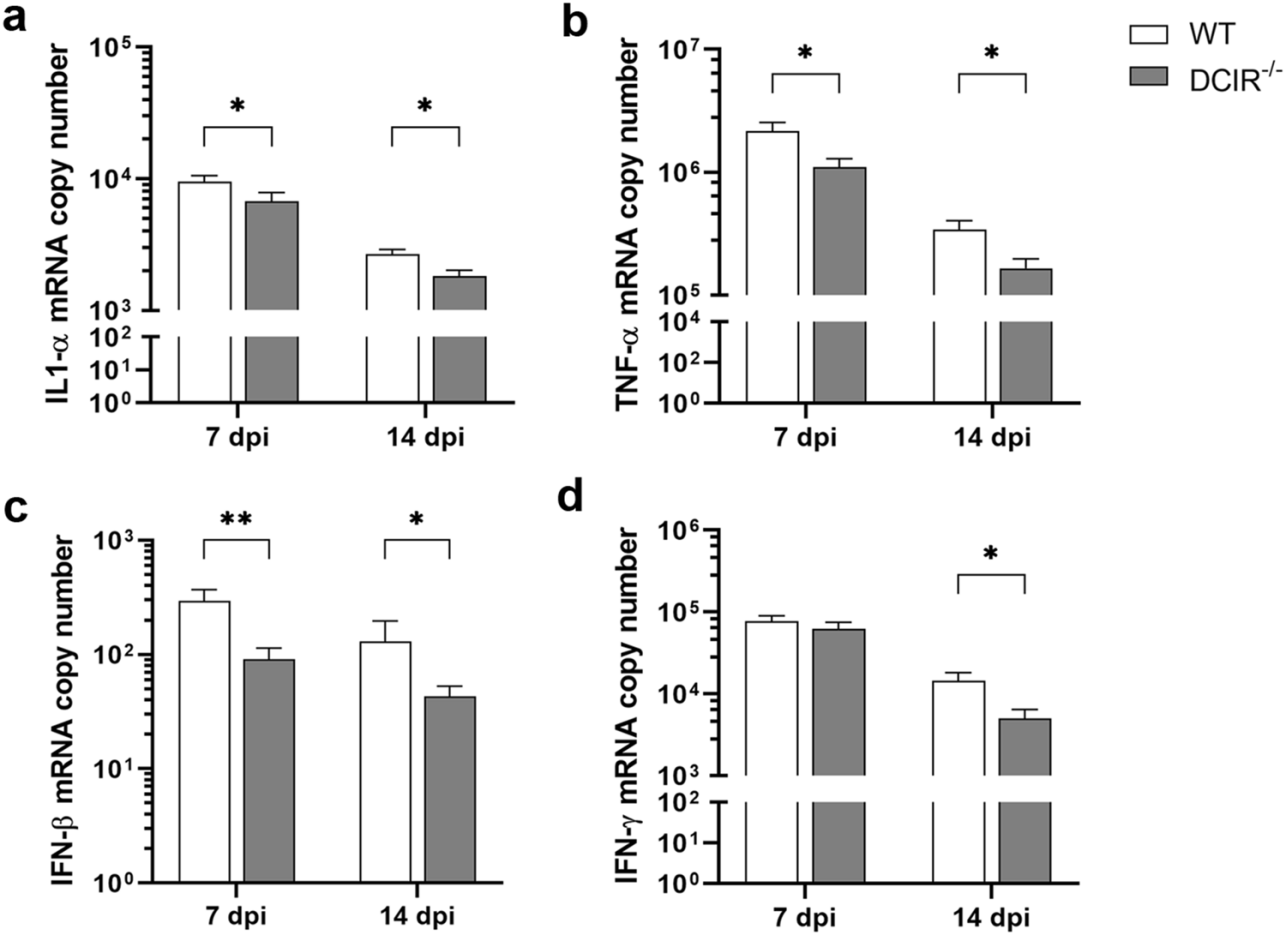
Cytokine expression within the cerebrum of mice infected with Theiler’s murine encephalomyelitis virus. Quantification of (**a**) interleukin (IL)-1α, (**b**) tumor necrosis factor (TNF)-α, (**c**) interferon (IFN)-β and (**d**) IFN-γ mRNA in the cerebrum of wild type (WT) and DCIR^−/−^ mice by reverse transcriptase quantitative polymerase chain reaction. (**a**–**d**) Statistical analysis: Mann–Whitney *U* test (statistical differences: *p ≤ 0.05, **p ≤ 0.01); data are shown as mean with SEM. n: 7 dpi = 9 WT and 12 DCIR^−/−^ mice; 14 dpi = 10 WT and 9 DCIR^−/−^ mice. *dpi * days post infection.

At 7 dpi, mRNA levels of IL-1α (*p* = 0.047, Fig. 4a), TNF-α (*p* = 0.039, Fig. 4b) and IFN-β (*p* = 0.009, Fig. 4c) were significantly reduced in TMEV-infected DCIR^−/−^ animals compared to the WT group. Data show a reduced pro-inflammatory response to virus infection due to receptor deficiency during the early acute polioencephalitis phase (7 dpi). In addition to mRNA levels of IL-1α (*p* = 0.018, Fig. 4a), TNF-α (*p* = 0.022, Fig. 4b), and IFN-β (*p* = 0.034, Fig. 4c), a significantly lower IFN-γ transcription was detected in DCIR^−/−^ animals compared to WT mice also at 14 dpi (*p* = 0.027, Fig. 4d). The mRNA levels of the pro-inflammatory cytokines IL-1β, IL-2, IL-6 and IL-23, as well as the anti-inflammatory cytokines IL-4, IL-5 and TGF-β1 did not differ significantly between both groups (Supplementary Fig. S2). Non-infected DCIR^−/−^ mice showed a significantly lower base line mRNA expression of IL-5 compared to WT mice (*p* = 0.021, Supplementary Fig. S5). Other cytokines mRNA levels of non-infected controls showed no differences between DCIR^−/−^ and WT animals (Supplementary Fig. S5). Statistical analyses (Pearson’s correlation coefficient R) revealed negative correlations between neuronal integrity of the hippocampus (NeuN^+^ area/mm^2^) and mRNA expression levels of IFN-β and IL-1β, and a positive correlation with IL-2 at 7 dpi. IFN-γ was negatively correlated with neuronal integrity at 14 dpi (Supplemental Table S6).

Reduced pro-inflammatory cytokine expression in the brain at the later stage of acute polioencephalitis (14 dpi) in DCIR^−/−^ mice is likely a direct consequence of reduced viral burden and accelerated termination of neuroinflammation in comparison to WT mice.

### Diminished induction of immunomodulatory responses in DCIR^−/−^ mice following neurotropic virus infection

**Figure 5:**
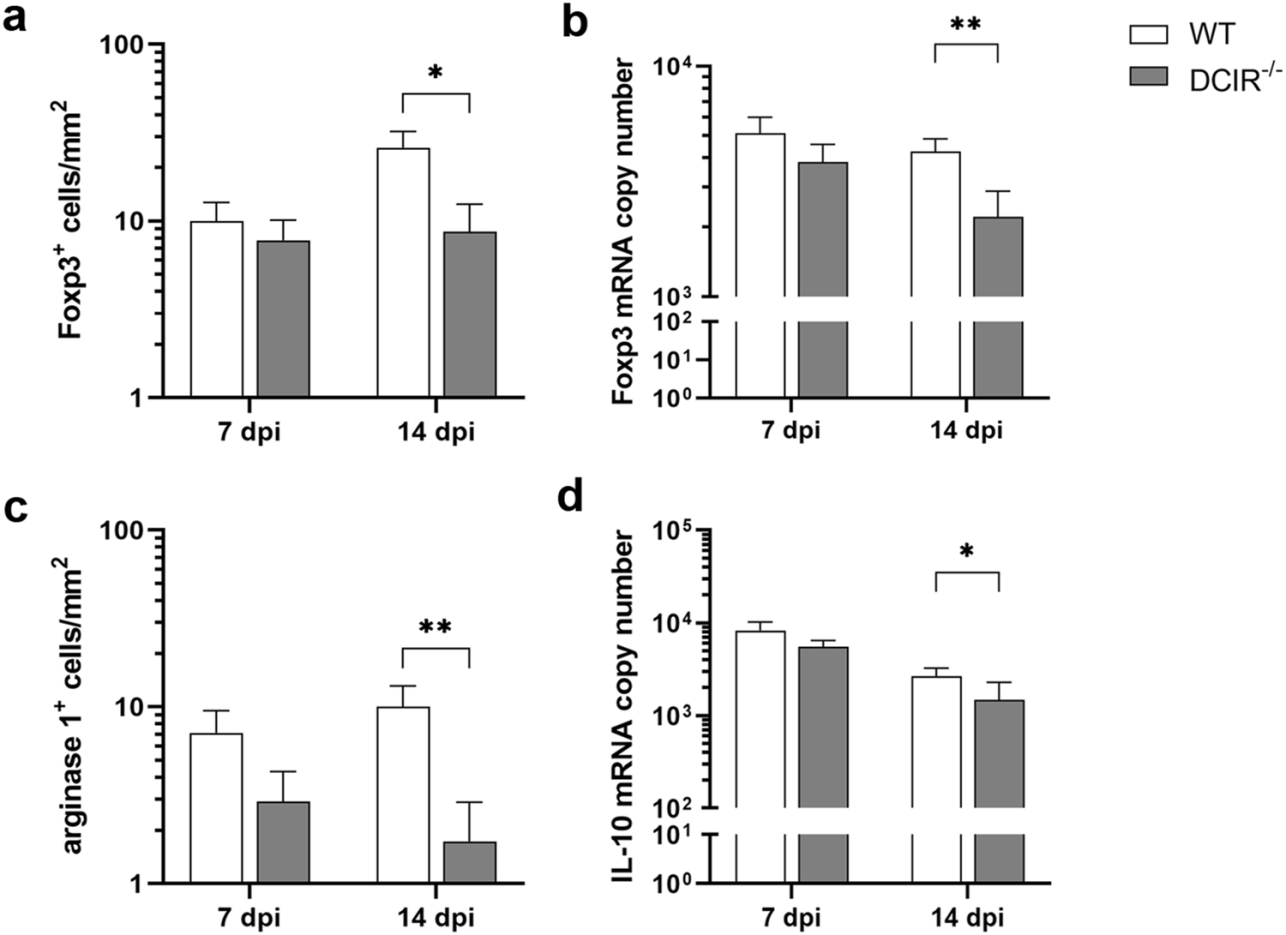
Quantification of arginase 1^+^ macrophages/microglia, Foxp3^+^ regulatory T cells, and interleukin 10 in the brain of Theiler’s murine encephalomyelitis virus infected mice. Quantification of (**a**) Foxp3^+^ regulatory T cells by immunohistochemistry, (**b**) Foxp3 mRNA transcription by reverse transcriptase quantitative polymerase chain reaction, (**c**) arginase 1^+^ M2-type macrophages/microglia by immunohistochemistry, (**d**) interleukin (IL)-10 mRNA transcription by reverse transcriptase quantitative polymerase chain reaction in wild type (WT) and DCIR^−/−^ mice. (**a**–**d**) Statistical analysis: Mann–Whitney *U* test (statistical differences: *p ≤ 0.05, **p ≤ 0.01); data are shown as mean with SEM. n: 7 dpi = 9 WT and 12 DCIR^−/−^ mice; 14 dpi = 10 WT and 9 DCIR^−/−^ mice. *dpi * days post infection.

The suppressive cytokine interleukin-10, secreted by regulatory T cells (Treg) and M2-type macrophages/microglia, is thought to exhibit neuroprotective effects in infectious disorders^40^. On the other hand, Treg and M2-type myeloid cells may dampen effective antiviral responses and thus promote deleterious effects on tissue integrity^41–43^. In order to test whether neuronal preservation in the hippocampus in DCIR^−/−^ mice is accompanied by reduced virus load or attributed to immunomodulatory mechanisms, Foxp3^+^ Treg, arginase 1^+^ M2-type macrophages/microglia and the key immunomodulatory cytokine IL-10 were quantified. At 14 dpi, numbers of Foxp3^+^ regulatory T cells (*p* = 0.018, Fig. 5a) and Foxp3 mRNA copy numbers (*p* = 0.009, Fig. 5b), determined by immunohistochemistry and RT-qPCR, respectively, were significantly increased in brain samples of mice with intact DCIR signalling (WT mice) compared to DCIR^−/−^ mice upon infection. Similarly, an increase of arginase 1^+^ cells was found in the hippocampus of WT mice compared to DCIR^−/−^ mice at both investigated time points with significant differences at 14 dpi (*p* = 0.006, Fig. 5c). Moreover, significantly increased transcription of IL-10 was detected in WT controls compared to DCIR^−/−^ mice at 14 dpi (*p* = 0.034, Fig. 5d). In non-infected WT and DCIR^−/−^ mice cerebral IL-10 mRNA expression was not detectable.

Results suggest that TMEV infection elicits compensatory immune pathways, mediated by Treg and M2-type macrophages/microglia in the brain of DCIR intact C57BL/6 mice. Consequently, the observed neuroprotective effect of DCIR deficiency is likely not mediated by classical immunomodulatory mechanisms at the infection site, but rather due to improved virus elimination and timely onset of peripheral protective immune responses. In addition, diminished induction of immunomodulatory and suppressive mechanisms, including decreased numbers of arginase 1^+^ M2-type macrophages/microglia and Treg, may promote virus control in DCIR deficient animals.

### DCIR deficiency enhances peripheral T cell responses following neurotropic virus infection

The accelerated TMEV elimination observed in the brain of DCIR^−/−^ mice suggests an enhanced antiviral immune response. Priming of naïve T cells in lymphoid organs during the early phase of TME was shown to be crucial for robust antiviral responses^44,45^. Therefore, splenic cytokine mRNA expression was quantified by RT-qPCR and splenic T cell responses were analysed by flow cytometry.

**Figure 6:**
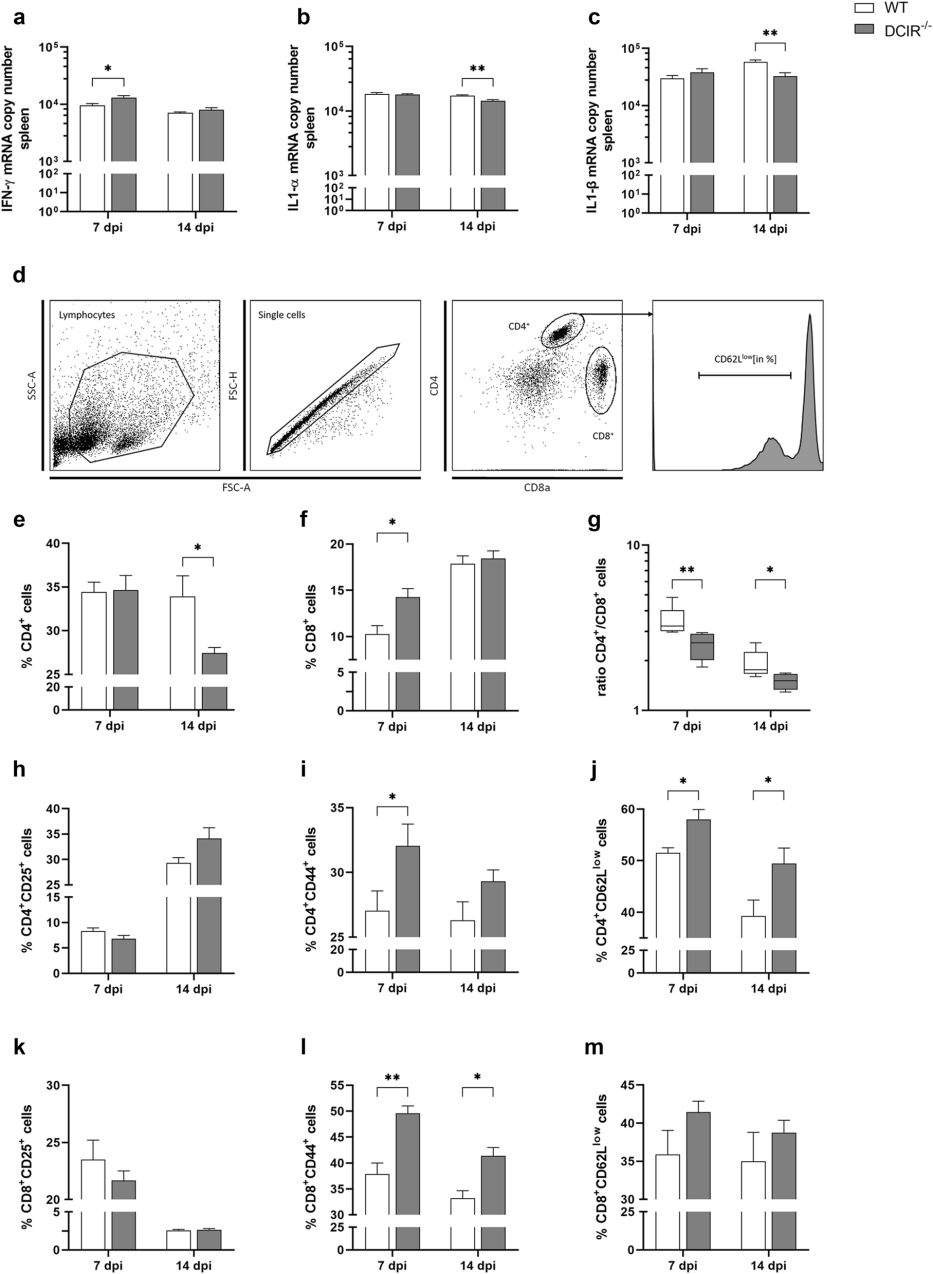
Splenic cytokine expression (**a**–**c**) and flow cytometric analysis of splenic T cells (**d**–**m**) in Theiler’s murine encephalomyelitis virus-infected DCIR^−/−^ and wild type (WT) mice. Quantification of (**a**) interferon (IFN)- γ, (**b**) interleukin (IL)-1α, (**c**) IL-1β mRNA in the spleen of wild type (WT) and DCIR^−/−^ mice by reverse transcriptase quantitative polymerase chain reaction. (**d**) Gating strategy of splenic T cells. Here, the gating for CD4^+^CD62L^low^ T helper cells is shown. The same procedure was used for all activation markers. Percentages of (**e**) CD4^+^ T helper cells, (**f**) CD8^+^ cytotoxic T cells, (**g**) ratio of CD4^+^ T helper cells to CD8^+^ cytotoxic T cells, (**h**) CD4^+^CD25^+^ T helper cells, (**i**) CD4^+^CD44^+^ T helper cells, (**j**) CD4^+^CD62L^low^ T helper cells, (**k**) CD8^+^CD25^+^ cytotoxic T cells, (**l**) CD8^+^CD44^+^ cytotoxic T cells and (**m**) CD8^+^CD62L^low^ cytotoxic T cells in spleen samples. (**a**–**c**, **e**–**m**) Statistical analysis: Mann–Whitney *U* test (statistical differences: *p ≤ 0.05, **p ≤ 0.01); data are shown as mean with SEM (**a**–**c**, **e**–**f**, **h**–**m**) and box plots display median with 5–95% percentiles (**g**). (**a**–**c**) n: 7 dpi = 9 WT and 12 DCIR^−/−^ mice; 14 dpi = 10 WT and 9 DCIR^−/−^ mice. (**e**–**m**) n: 5 mice per group and time point. *dpi * days post infection.

At 7 dpi, splenic IFN-γ mRNA levels were significantly increased in TMEV-infected DCIR^−/−^ animals compared to the WT group (*p* = 0.049, Fig. 6a), indicating an enhanced pro-inflammatory immune response. At 14 dpi, IL-1α (*p* = 0.006, Fig. 6b) and IL-1β (*p* = 0.001, Fig. 6c) mRNA expression was reduced in TMEV-infected DCIR^−/−^ animals compared to WT mice, likely related to the reduced viral burden and accelerated termination of neuroinflammation in DCIR deficient mice. Splenic IL-2, IL-4, IL-5, IL-6, IL-10, IL-23, IFN-β, TGFβ1, and TNF-α mRNA levels did not differ significantly between both groups (Supplementary Fig. S3).

Flow cytometric analysis of TMEV-infected groups revealed an increased fraction of CD8^+^ T cells in the spleen of DCIR^−/−^ mice compared to WT group at 7 dpi (*p* = 0.016, Fig. 6f), while CD4^+^ T cell frequency remained unchanged at this time point (Fig. 6e). Accordingly, a significant shift of the CD4^+^/CD8^+^ T cell ratio with dominance of cytotoxic T cells was observed at 7 dpi (*p* = 0.009, Fig. 6g) as well as at 14 dpi (*p* = 0.028, Fig. 6g). Subsequently, the portion of splenic CD4^+^ T cells decreased in DCIR^−/−^ mice during the disease course, resulting in a significant difference between the groups at 14 dpi (*p* = 0.028, Fig. 6e).

Further characterization of splenic T cell subsets revealed a significantly higher proportion of activated CD4^+^ T cells, displayed by higher fractions of CD4^+^CD62L^low^ T cells in DCIR^−/−^ mice compared to WT mice at 7 dpi (*p* = 0.016) and 14 dpi (*p* = 0.047, Fig. 6d,j). Moreover, the level of activated CD4^+^CD44^+^ T cells was elevated at both time points in DCIR^−/−^ animals compared to WT mice with statistically significant difference between the groups at 7 dpi (*p* = 0.047, Fig. 6i). Similarly, CD8^+^CD44^+^ cell populations were significantly increased in DCIR^−/−^ animals compared to WT mice at 7 dpi (*p* = 0.009) and 14 dpi (*p* = 0.016, Fig. 6l). Splenic CD4^+^- and CD8^+^ T cell subpopulations expressing CD25 as well as CD8^+^CD62L^low^ T lymphocytes did not significantly differ between groups at any time point (Fig. 6h,k,m). Flow cytometry of non-infected control mice showed a slight difference for the portion of CD8^+^ T cells and the CD4^+^/CD8^+^ T cell ratio comparing the spleens of WT and DCIR^−/−^ mice. However, no major differences in surface expression of T cell activation markers were observed between both non-infected control groups (Supplementary Fig. S6). Thus, flow cytometry and cytokine expression analysis revealed an enhanced peripheral T cell activation and an early proportional shift towards cytotoxic CD8^+^ T cell responses in DCIR^−/−^ mice during viral encephalitis.

### Identification of potential influencing factors for hippocampal damage using regression analyses

Regression analyses were performed to identify factors that correlate with hippocampal damage following TMEV infection. Simple regression models confirmed that neuronal integrity of the hippocampus (NeuN^+^ area/mm^2^) was significantly associated with the TMEV load. In addition, hippocampal damage was significantly associated with the amount of CD107b^+^, CD3^+^, CD45R^+^ Foxp3^+^, arginase 1^+^, GFAP^+^, and granzyme B^+^ cells in the hippocampus, as well as with IFN-β mRNA expression in the brain. The amount of TMEV^+^ cells in the hippocampus remains the only significant parameter in the multiple regression model, pointing at a high collinearity among explanatory variables (Supplementary Table S5).

### Reduced neuroinflammation in DCIR^−/−^ mice following neurotropic virus infection correlates with diminished responses of antigen presenting cells

**Figure 7:**
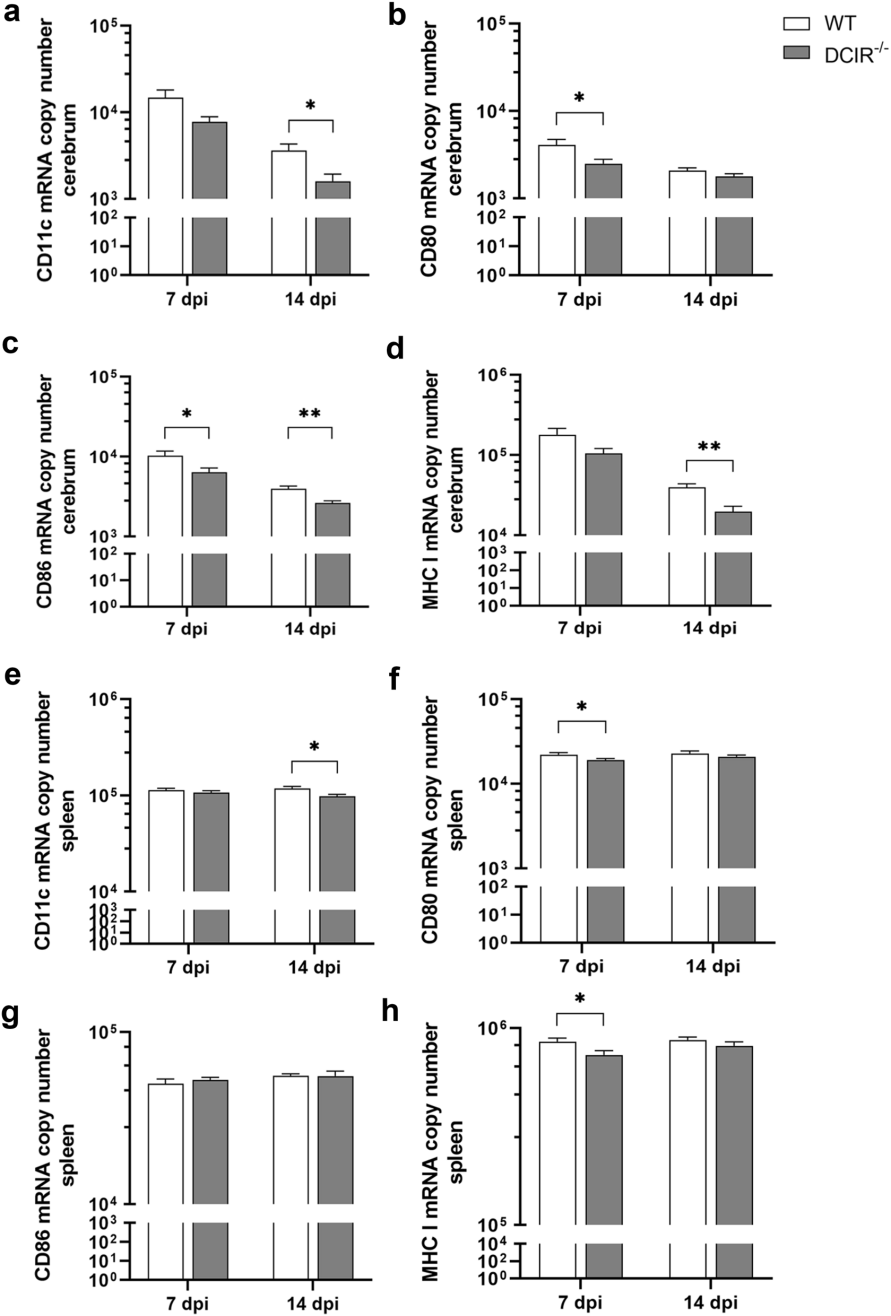
Analysis of CD11c, CD80, CD86 and MHC-I mRNA expression within the cerebrum and spleen of Theiler’s murine encephalomyelitis virus-infected DCIR^−/−^ and wild type (WT) mice. Quantification of (**a**) CD11c, (**b**) CD80, (**c**) CD86, and (**d**) MHC-I mRNA level within the cerebrum and of (**e**) CD11c, (**f**) CD80, (**g**) CD86 and (**h**) MHC-I mRNA expression within the spleen by reverse transcriptase quantitative polymerase chain reaction. (**a**–**h**) Statistical analysis: Mann–Whitney *U* test (statistical differences: *p ≤ 0.05, **p ≤ 0.01); data are shown as mean with SEM. n: 7 dpi = 9 WT and 12 DCIR^−/−^ mice; 14 dpi = 10 WT and 9 DCIR^−/−^ mice. *dpi * days post infection.

At 14 dpi, significantly reduced CD11c mRNA copy numbers were detected within the brains (*p* = 0.017, Fig. 7a) and spleens of DCIR^−/−^ mice (*p* = 0.035, Fig. 7e). Moreover, CD80 mRNA expression levels were significantly reduced within the brains (*p* = 0.049, Fig. 7b) and spleens (*p* = 0.034, Fig. 7f) of DCIR^−/−^ mice in comparison to WT animals at 7 dpi. CD86 mRNA transcript levels were significantly diminished within the brains of DCIR^−/−^ mice in comparison to WT mice at both time points (7 dpi: *p* = 0.049, 14 dpi: *p* = 0.001, Fig. 7c). Within the spleen, CD86 mRNA transcript levels showed no differences between DCIR^−/−^ and WT animals (Fig. 7g). MHC-I mRNA expression was significantly reduced in spleens of DCIR deficient mice at 7 dpi (*p* = 0.049, Fig. 7h), whereas cerebral MHC-I mRNA quantities were significantly decreased in DCIR^−/−^ mice compared to WT animals at 14 dpi (*p* = 0.003, Fig. 7d). Reduced expression of CD11c and co-stimulatory molecules in the brains and spleens of DCIR^−/−^ mice may be linked to the accelerated resolution of TMEV infection and termination of neuroinflammatory response in comparison to WT mice.

### Lack of DCIR in bone marrow-derived dendritic cells causes an increased CD8^+^ T cell response against Theiler’s murine encephalomyelitis virus in vitro

**Figure 8:**
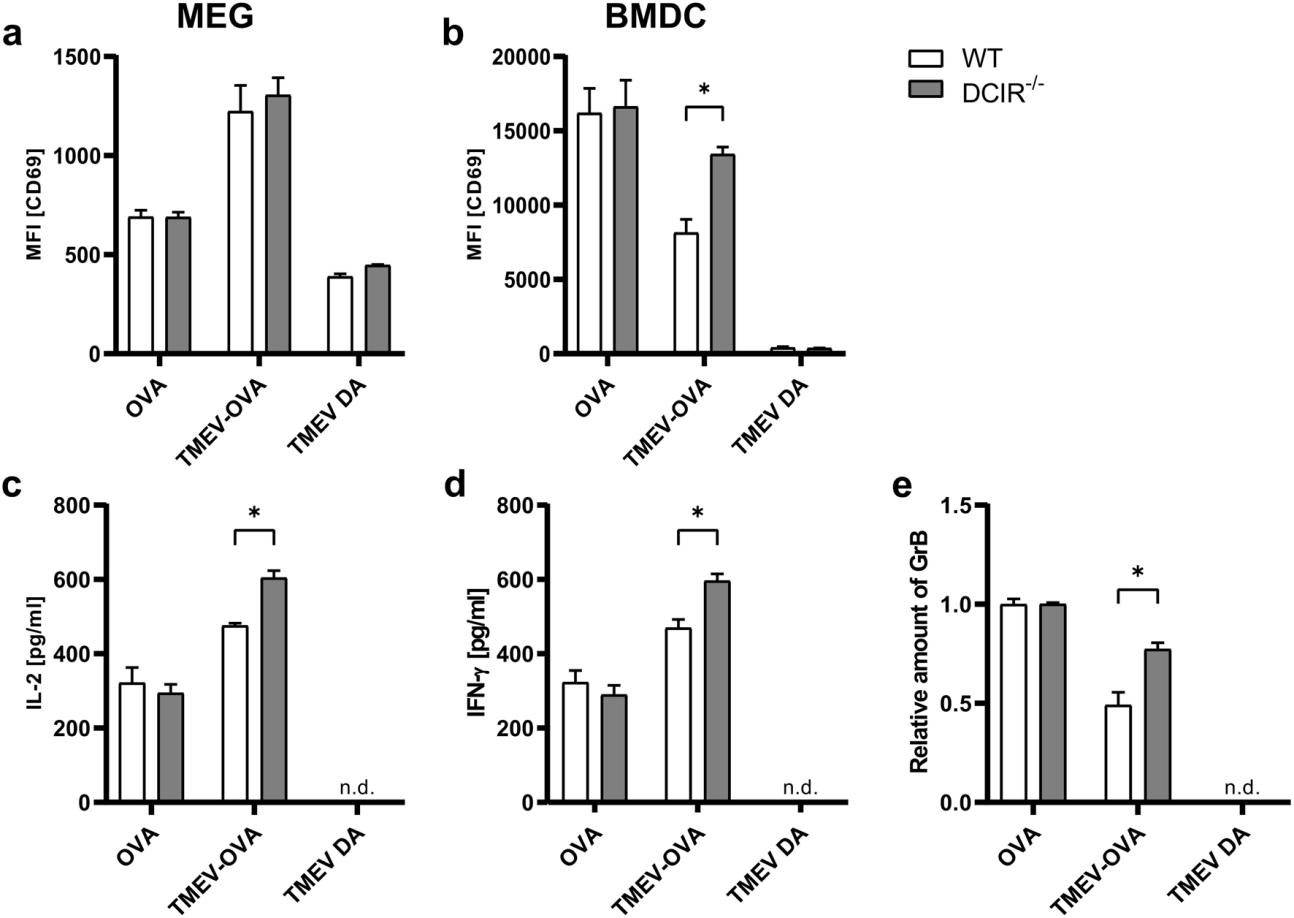
CD8^+^ T cell response induced by pulsing of microglia-enriched glial cell mixtures (MEGs) or bone marrow-derived dendritic cells (BMDCs) with Theiler’s murine encephalomyelitis virus Daniels strain XhoI-OVA8 (TMEV-OVA) in vitro. Flow cytometric assessment of mean fluorescence intensity (MFI) of activation marker CD69 gated on CD8^+^ cytotoxic T cells derived from OT-I mice after co-cultivation with (**a**) MEGs and (**b**) BMDCs**.** Analysis of the cytokines (**c**) interleukin 2 (IL-2), (**d**) interferon γ (IFN-γ) and (**e**) granzyme B (GrB) by ELISA following co-cultivation with BMDCs. (**a**–**e**) Statistical analysis: Mann–Whitney *U* test (statistical differences: *p ≤ 0.05); data are shown as mean with SEM. n.d. = not detectable. (**a**–**d**) Representative for n = 3. (**e**) Relative amount of granzyme B compared to OVA control, representative for n = 2.

MHC-I-restricted CD8^+^ cytotoxic T cells are important for TMEV elimination in C57BL/6 mice^46^. To determine the impact of DCIR deficiency on early T cell responses upon TMEV infection in vitro*,* antigen presentation assays using WT and DCIR^−/−^ MEGs or BMDCs were performed. T cells were isolated from OT-I TCR-transgenic mice, which specifically recognise the OVA-peptide presented via the MHC-I molecule H2-K^b^. T cells were co-cultured with MEGs or BMDCs, previously exposed to TMEV-OVA^47–49^. BMDCs were used for the in vitro stimulation of OT-I T cells since BMDCs from WT and DCIR^−/−^ mice had been previously compared in a global and unbiased manner through genome-wide transcriptome analysis and thus represent a well characterized source of APCs^36^. To analyse CD8^+^ T cell activation, cytokine release was measured by ELISA, and expression of the early T cell activation marker CD69 was measured by flow cytometry. Microglia, as part of the glial cell mixtures, represent the CNS’ local APC population. Incubating MEGs with TMEV-OVA, however, did not result in a difference between WT or DCIR^−/−^ microglia-mediated CD8^+^ T cell response (Fig. 8a). Combined with the marginal levels of released pro-inflammatory cytokines (data not shown), these findings suggest that the potential of microglia to process and present antigens is limited. As an additional source of APCs, BMDCs were used in a co-culture system to stimulate antigen-specific CD8^+^ T cells. To avoid alterations in TMEV antigenicity and modification of (potential) DCIR ligands, live virus was used for incubation with BMDCs. However, to exclude a productive infection of BMDCs leading to classical antigen presentation via MHC-I molecules, viral RNA loads in TMEV DA-exposed BMDCs as well as viral titers in the supernatant were determined (Supplementary Fig. S7). While an initial increase in viral RNA load in BMDCs between 2 and 6 h was observed (Supplementary Fig. S7a), viral titers in the supernatant decreased continuously from 2 to 22 h after TMEV DA incubation (Supplementary Fig. 7b). Thus, the initial increase of TMEV RNA in BMDCs may be mediated by initial TMEV replication, but it most likely does not reflect a productive infection of BMDCs, but rather an increased TMEV internalisation. In addition, incubation with live TMEV did not lead to a significant decrease in MEG and BMDC viability compared to OVA- or mock-stimulated samples and the vast majority of the cells remained viable (Supplementary Fig. S8), further supporting that BMDCs present viral antigens to CD8^+^ T cells. Upon TMEV DA incubation, BMDCs were activated, but no difference between WT and DCIR^−/−^ BMDCs was detected (Supplementary Fig. S9). Similarly, the activation status of BMDCs did not differ between WT and DCIR^−/−^ BMDCs following co-cultivation with OT-I T cells (Supplementary Fig. S10). However, TMEV-OVA stimulation of DCIR^−/−^ BMDCs led to an increased expression of CD69 by CD8^+^ T cells compared to WT BMDCs (Fig. 8b). Further, the release of IL-2, IFN-γ and GrB by CD8^+^ T cells was elevated if co-cultured with DCIR^−/−^ BMDCs (Fig. 8c–e). These results indicate that DCIR deficiency in BMDCs impacts subsequent CD8^+^ T cell activation and T cell effector functions in this BMDC/T cell co-culture system. Possibly, DCIR in DCs may balance type I and II IFN signaling directly influencing T cell priming^36,50^. Additionally, cross-talk of DCIR with other immune receptors, such as Toll-like receptors, is conceivable, which can affect the quality of induced T cell responses even without alterations in the expression of co-stimulatory markers CD80 and CD86, as it was shown for human DCIR^51–53^. However, the mechanism by which the differential CD8^+^ T cell activation by DCIR^−/−^ BMDCs shown here is mediated, remains to be determined in future studies.

## Discussion

This study highlights the role of DCIR in neuropathology of C57BL/6 mice following acute TMEV infection. Genetic ablation of DCIR appears to exert a supporting effect on viral clearance from the CNS and ameliorates hippocampal damage following virus infection.

While susceptible mouse strains (e.g. SJL mice) show an inefficient antiviral immunity and persistent TMEV infection in the CNS, C57BL/6 mice develop vigorous TMEV-specific responses during acute infection^54^. The ability of C57BL/6 mice to eliminate TMEV is caused by robust MHC class I-restricted antiviral CD8^+^ T cell responses^39,40,46,55–57^. As shown in the present study, the lack of DCIR contributes to a more effective priming of peripheral T cells with increased CD44 and reduced CD62L expression by CD4^+^ T cells together with an increased IFN-γ expression in the spleen during the early phase of TMEV infection. In general, DCIR^−/−^ mice show an age-related increase of CD4^+^CD44^high^ and CD4^+^CD62^low^ T cells by expanding DC populations in lymphoid organs, demonstrating that DCIR deficiency predisposes to effector-memory T cell development. CD4^+^ T cells are required for protective immunity in TMEV infection, since CD4 deficiency has been shown to cause virus persistence in C57BL/6 mice. CD4^+^ helper T cells support antiviral CD8^+^ T cell responses by cytokine release (e.g. IL-2) and by improving the ability of DCs to prime cytotoxic T cell responses (DC licensing)^33,58,59^. An increased frequency of splenic CD8^+^ T cells together with an upregulation of the activation marker CD44 was found in infected DCIR^−/−^ mice, suggesting an enhancement of cytotoxic CD8^+^ T cell responses. The skewed ratio of CD4^+^ to CD8^+^ T cells observed in DCIR^−/−^ mice indicates an early dominance of peripheral cytotoxic responses. Increased frequencies of circulating CD8^+^ T cells were shown to improve antiviral immunity and account for TMEV elimination in C57BL/6 mice^39,60^. In agreement with the present findings, enhanced T cell responses in DCIR^−/−^ mice control experimental mycobacteria infection better than WT controls. Of note, DCs of DCIR^−/−^ mice exhibit several transcriptional changes that promote Th1 immunity also under non-infectious conditions^36^.

Noteworthy, besides protecting from viral infection, T cell immunity has the ability to contribute to acute brain pathology following TMEV infection. Virus-specific CD8^+^ T cells target infected neurons of the hippocampus in acutely infected C57BL/6 mice. MHC class I-restricted cytotoxicity towards TMEV epitopes contributes to neuronal loss and brain atrophy^57,61,62^. Moreover, cytotoxicity boosted by TMEV peptides leads to fatal CNS inflammation in infected C57BL/6 mice, demonstrating the difficulty of balancing immune responses in neurotropic virus infection^62,63^. As observed in experimental autoimmune encephalomyelitis, rheumatoid arthritis models, and experimental colitis, DCIR^−/−^ mice are prone to develop autoimmunity and T cell-mediated immunopathology, respectively^33,64,65^. Strikingly, despite enhanced peripheral cytotoxic responses in the present study, no exacerbated brain injury was observed in DCIR^−/−^ mice, but on the contrary, a reduced hippocampal damage following TMEV infection. DCIR deficiency seems to fine-tune protective immune responses without evoking additional virus-mediated immunopathology in the TME model. The underlying mechanisms remain speculative, but might be associated with diminished pro-inflammatory cytokine responses found in the brain of DCIR^−/−^ mice. Reduced expression of IFN-β and TNF-α in the brain of DCIR^−/−^ mice during the early phase of polioencephalitis (7 dpi) indicates a diminished cytokine response at the infection site. Particularly, increased IFN-β mRNA levels were significantly associated with hippocampal damage in TMEV-infected mice as determined by correlation analyses. IFN-β (type I interferon) expression in the brain is driven by TMEV infection and involved in the induction of innate and adaptive immune responses^66^. Robust antiviral immunity trigged by type I interferons accounts for viral elimination in C57BL/6 mice but also elicit neuronal damage following TMEV infection^10^. Thus, reduced IFN-β expression might have contributed to diminished T cell sequestration in the brain and decreased hippocampal damage in DCIR^−/−^ mice during advanced infection (14 dpi). Similarly, TNF-α is a cytokine produced by activated microglia and macrophages, which initiate protective responses against certain viral infections, including TMEV infection^67,68^. However, TNF-α also displays cytotoxic effects and contributes to hippocampal damage in C57BL/6 mice following TMEV infection^7,10,20^. In addition, TNF-α has been shown to cause excitotoxicity and neuronal damage in murine HIV encephalitis models^69^. Thus, in addition to the accelerated virus elimination, the alleviated brain cytokine response at the infection site might also contribute to the neuroprotective effect observed in DCIR^−/−^ mice. Despite differences of hippocampal integrity and cytokine expression profiles, no obvious clinical changes were observed between DCIR^−/−^ mice and WT mice (subclinical infection). More targeted diagnostic methods such as video/EEG monitoring and behavioral tests (e.g. Morris water maze) are needed to detect subtle clinical changes and fully discover the functional relevance CNS alteration in receptor deficient animals in future studies.

In addition to an enhanced CD8^+^ T cell activation in the periphery during the early infection phase, an altered immune environment at the site of infection, including reduced infiltrations of Foxp3^+^ Treg and arginase 1^+^ M2-type cells in DCIR^−/−^ animals at 14 dpi might have influenced TMEV control. Consistent with this, a decreased expression of genes specific for M2-type cells (including arginase 1) can be found in DCIR^−/−^ mice following mycobacteria infection^36^. Moreover, arginase 1^+^ myeloid cells have been shown to exert suppressive effects on antiviral immunity^42^. For instance, ablation of arginase 1 in macrophages reduces the viral load and ameliorates tissue integrity after experimental Ross River virus infection of mice^43^. Similarly, Treg are able to dampen antiviral responses during TMEV infection^39^.

The interplay between innate and adaptive immunity is mediated by APCs, such as macrophages, microglia and DCs, which have the ability to recognize pathogens and induce effector T cell responses^51,70,71^. Microglia are CNS-resident APCs and play an important role in TMEV-mediated hippocampal damage and seizure development^17^. However, within the present study, in vitro TMEV exposure of DCIR^−/−^ and WT MEGs did not show differences in CD8^+^ T cell activation. Although there was a slight OVA- and TMEV-OVA-mediated increase of CD69 observed in the MEG/T cell co-cultivation assay, cytokine levels were not elevated. Thus, in comparison to DCs, the in vitro potential of adult microglia to perform APC function and present specific antigens is apparently limited, as previously shown^72,73^.

DCIR is expressed on all DC subsets and exerts mainly inhibitory effects on immune responses via its intracellular ITIM^28,30,33,50,74,75^. The present study shows an enhanced activation of CD8^+^ T cells when DCIR^−/−^ BMDCs were used to prime CD8^+^ T cells. In addition, the release of IL-2 by activated T cells was elevated upon co-cultivation with DCIR^−/−^ BMDCs. DCIR deficiency results also in an increased production of IFN-γ by lymphocytes which was also observed in the present study^50^. Likewise, Chikungunya virus infection of DCIR^−/−^ mice causes an elevation of IFN-γ in vivo^37^. However, in contrast to the TME model, intact DCIR signalling in experimental Chikungunya virus infection contributes to protection against virus-induced pathology of the joint, demonstrating that the effect of DCIR signalling on disease progression is clearly context dependent and differs between pathogens and the primarily affected organ in infectious disorders^37^.

Conclusively, DCIR deficiency seems to support antiviral immune responses of C57BL/6 mice during the initial phase of TMEV infection and to reduce virus-induced neuropathology. Previous studies highlight the potential of DCIR for cell specific targeting and immune modulation^51,52,76^. Thus, this CLR represents a potential target for intervention strategies to selectively enhance protective immunity in neurotropic virus infection.

## Material and methods

### Animals

DCIR^−/−^ mice (C57BL/6-Clec4a2^tm1.1Cfg^/Mmucd; RRID:MMRRC_031932-UCD) were obtained from the National Institutes of Health-sponsored Mutant Mouse Resource & Research Center (MMRRC) National System^38^. The mouse strain was backcrossed on C57BL/6 background over more than ten generations^38^. DCIR^−/−^ and respective C57BL/6 mice (WT) were used for the infection experiment. All mice were housed in the animal facility of the University of Veterinary Medicine (Hannover, Germany) in individually ventilated cages under controlled conditions (12 h light/12 h dark cycle, 22–24 °C, humidity 50–60%) with permanent access to water and standard rodent feed. Animal experiments were conducted in accordance with the German law for animal protection and the Directive 2010/63/EU of the European Parliament and of the Council on the protection of animals used for scientific purposes and the ARRIVE guidelines^77^. The study was approved and authorized by the Niedersächsisches Landesamt für Verbraucherschutz und Lebensmittelsicherheit (LAVES), Oldenburg, Germany (permission number 33.19-42502-04-16/2225, date of approval: October 7, 2016).

### Virus and cell lines

For intracerebral injection, the Daniels strain of TMEV (TMEV DA) was used^78^. TMEV DA and live ovalbumin (OVA) peptide-expressing TMEV DA XhoI-OVA8 (TMEV-OVA) were utilized for in vitro bone marrow-derived dendritic cell (BMDC) and adult microglia-enriched glial cell mixtures (MEG)/T cell co-cultivation assays. TMEV-OVA was generated by integrating the coding sequence corresponding to the amino acid sequence OVA_(251–267)_ of chicken egg albumin. Flanking sequences were included to assure natural processing of the immunodominant H-2 Kb restricted epitope OVA(_257–264_; SIINFEKL). Previous use of this virus has demonstrated viral replication in the CNS of intracranial infected C57BL/6 mice^49^. Furthermore this live virus vector has demonstrated robust generation of H-2 Kb restricted CD8^+^ T cell responses to the OVA_(257–264)_ antigen after infection and in tumor models^47–49^. Virus strains were cultivated and passaged in BHK-21 cells and plaque assays were performed using L-cells for virus titration^79–81^.Virus isolation was performed by freezing and thawing. Plaque assays were performed as independent duplicates. Virus titres were determined by calculating the plaque forming units per ml (PFU/ml) as previously described^82,83^.

### Experimental design

Five-week old female DCIR^−/−^ and WT mice were anaesthetised with medetomidine (1 mg/kg, Domitor) and ketamine (100 mg/kg) and inoculated into the right cerebral hemisphere with TMEV DA in a total volume of 20 µl DMEM (Biochrom GmbH, Berlin, Germany) supplemented with 2% FCS (PAA Laboratories GmbH, Pasching, Austria) and 50 µg/kg gentamicin (Sigma Aldrich Chemie GmbH, Taufkirchen, Germany) as described^39^. Weekly clinical examination included body weight recordings as well as clinical scorings with evaluation of “posture and outer appearance”, “behaviour and activity” and “gait”^39^. Additionally, a 5 point scale scoring system according to Racine (Racine score) was applied for recording motor seizures^84^. A RotaRod (TSE Systems GmbH, Bad Homburg, Germany) performance test for motor function and coordination was carried out weekly^85^.

At 7 and 14 days post infection (dpi) mice were anaesthetised as described above and euthanised with an overdose of medetomidine (1 mg/kg) and ketamine (200 mg/kg). The rostral part of the left cerebrum (contralateral to injection site) was formalin fixed and paraffin embedded (FFPE), and caudal part of the left cerebrum was snap frozen and stored at − 80 °C^39,86^. Spleens were taken for flow cytometry and parts of splenic tissue were snap frozen and stored at − 80 °C. Serial sections (2–3 µm thickness) of FFPE coronal brain sections at the hippocampal level (Bregma − 1.46 to − 1.82) were used for histology (hematoxylin and eosin staining) and immunohistochemistry, respectively^39,87^. In addition, non-infected age matched controls were used to determine baseline differences of splenic and hippocampal immune cell compositions as well as cerebral cytokine and transcription factor expression profiles between DCIR^−/−^ and WT mice.

### Histologic scoring of hippocampal lesions

Hippocampal damage was evaluated using a semiquantitative scoring system, assessing the integrity of the pyramidal neurons: score 0 = no obvious damage; score 1 = loss involving < 10% of neurons; score 2 = loss involving < 20% of neurons; score 3 = loss involving 20–50% of neurons; score 4 = loss involving > 50% of neurons^88^.

### Immunohistochemistry

Immunohistochemistry was used to detect macrophages/microglia (CD107b, arginase 1), T cells (CD3, CD4, CD8), B cells (CD45R), granzyme B (GrB), regulatory T cells (Foxp3), neurons (neuronal nuclei, NeuN), axons (β-amyloid precursor protein, β-APP), astrocytes (glial fibrillary acidic protein, GFAP), and TMEV capsid protein VP1 as described^89–91^. Used antibodies and staining procedures are listed in Supplementary Table S1. Primary antibodies were diluted in PBS including 1% bovine serum albumin (BSA). In brief, endogenous peroxidase was inhibited by 0.5% H_2_O_2_ in ethanol for 30 min. For antigen retrieval (CD107b, arginase 1, CD3, CD45R, Foxp3, NeuN, GrB, β-APP), slides were incubated in citrate buffer within a microwave oven for 20 min. Blocking of unspecific bindings was conducted with either goat serum (TMEV, arginase 1, CD3, NeuN, GrB, β-APP, GFAP) or rabbit serum (CD107b, Foxp3, CD4, CD8). Following, primary antibodies were incubated over night at 4 °C. Biotinylated goat anti-rabbit IgG antibody was used as secondary antibody for TMEV-, arginase 1-, CD3-, and GFAP-specific immunohistochemistry. For CD107b-, CD4-, CD8-, and Foxp3-specific staining, a biotinylated rabbit anti-rat IgG antibody was utilised, and a biotinylated goat anti-mouse IgG antibody was used for NeuN- and β-APP-specific staining. Slides were incubated with the avidin–biotin-peroxidase complex. For visualisation, slides were incubated with 3.3-diaminobenzidine-tetrahydrochloride in PBS containing 0.125% H_2_O_2_ and counterstained with Mayer’s hematoxylin.

Hippocampi were digitalised and measured by using the bright field mode of the fluorescence microscope BZ-9000 BIOREVO (HS All-in-one fluorescence microscope, Keyence Corporation, Osaka, Japan) and BZ-II Analyzer software (BZ-H2AE, Keyence Corporation, Osaka, Japan). For CD3-, CD107b-, TMEV-, NeuN- and GFAP-specific immunohistochemistry, the proportion of immunolabelled area within the hippocampus was quantified by densitometric analysis. Moreover, for quantifying TMEV-infected cells, arginase 1^+^ macrophages/microglia, CD45R^+^ B cells, Foxp3^+^ regulatory T cells, CD4^+^ T helper cells, CD8^+^ cytotoxic T cells, and GrB^+^ effector cells, absolute numbers of labelled cells within the hippocampus were counted (cells/mm^2^). In addition to densitometric analysis, neuronal loss (NeuN) was graded semiquantitavely as described above, too^88,92^. Evaluation of axonal damage (β-APP) was performed by a semiquantitative scoring system^93^. Axonal damage in the hippocampus was graded as followed: score 0 = no β-APP^+^ axons; score 1 = 1–25 β-APP^+^ axons; score 2 = 26–50 β-APP^+^ axons; score 3 = 51–75 β-APP^+^ axons; score 4 = 76–100 β-APP^+^ axons; score 5 = more than 100 β-APP^+^ damaged axons^93^.

### Ribonucleic acid isolation and reverse transcription

For RNA isolation, snap frozen tissue of the cerebrum was homogenized in 1 ml QIAzol lysis reagent (Qiagen, Hilden, Germany) with Omni Tip PCR Tissue Homogenizing Kit (Süd-Laborbedarf GmbH, Gauting, Germany). Subsequently, homogenates were treated with RNeasy Lipid tissue Mini Kit (Qiagen, Hilden, Germany) according to the manufacturer’s protocol. Likewise, RNA isolation of snap frozen splenic tissue has been performed using RNeasy Mini Kit (Qiagen, Hilden, Germany) according to the manufacturer’s protocol. The purity and amount of RNA was measured with a Multiskan GO microplate spectrophotometer (Thermo Fisher Scientific, Waltham, MA, USA) using a µDrop plate (Thermo Fisher Scientific, Waltham, MA, USA) and SkanIt software (version 3.2.1.4 RE, Thermo Fisher Scientific, Waltham, MA, USA)^94^. Equal amounts of RNA were translated into cDNA using Omniscript Reverse Transcription Kit (Qiagen, Hilden, Germany), RNaseOUT Recombinant Ribonuclease Inhibitor (Invitrogen, Thermo Fisher Scientific, Waltham, MA, USA) and random primers (Promega Corporation, Madison, WI, USA).

### Reverse transcription-quantitative polymerase chain reaction (RT-qPCR)

To determine viral load (TMEV RNA) and mRNA expression levels of CD11c, CD80, CD86, Foxp3, interleukin (IL)-1α, IL-1β, IL-2, IL-4, IL-5, IL-6, IL-10, IL-23, interferon (IFN)-β, IFN-γ, MHC-I, tumor necrosis factor (TNF)-α, transforming growth factor (TGF)-β1, and the three housekeeping genes, β-actin, glyceraldehyde 3-phosphate dehydrogenase (GAPDH), and hypoxanthine–guanine phosphoribosyltransferase (HPRT), RT-qPCR was carried out using the Mx3005P Multiplex Quantitative PCR System (Agilent Technologies Deutschland GmbH, Waldbronn, Germany) and Brilliant III Ultra-Fast SYBR Green QPCR Mastermix (Agilent Technologies Deutschland GmbH, Waldbronn, Germany). Primer details are listed in Supplementary Table S2. Quantification of copy numbers was achieved by parallel, duplicate amplification of tenfold serial dilution of standards ranging from 10^8^ to 10^2^ copies/µl. Melting curve analysis proved specificity of each reaction^86^. The geNorm software (version 3.4) was utilised for normalisation of qPCR data^95,96^.

### Flow cytometry of murine splenocytes

Spleens were removed and immediately flushed mechanically with a syringe and 1 × PBS to a single cell suspension. Subsequently erythrocytes were lysed using RBC lysis buffer (10% 100 mM Tris–HCl [Tris-(hydroxymethyl)-aminomethanhydrochloride], 90% 160 mM NH_4_Cl [ammonium chloride], Carl Roth, Karlsruhe, Germany). Afterwards, cells were incubated with rat-anti CD16/32 monoclonal antibody (1:100) to block the Fc gamma receptor and therefore to avoid unspecific binding. Cell solutions were stained with following monoclonal anti-mouse antibodies: CD4-FITC, CD62L-PE-Cy7, CD4-PerCP-Cy5.5, CD25-FITC, CD44-APC, CD8a-PE, CD8a-APC and CD19-FITC. Details for all flow cytometry antibodies are listed in Supplementary Table S3. For fixation, cells were incubated with 1% paraformaldehyde (PFA, Carl Roth, Karlsruhe, Germany). Flow cytometry was performed at the Attune NxT cytometer (Thermo Fisher Scientific, Waltham, MA, USA). Data analysis was conducted with FlowJo software (version 10, FloJo LLC, Ashland, OR, USA)^97^.

### Isolation of an adult microglia-enriched glial cell mixture (MEG)

To isolate MEGs, a previously used method was modified^98^. Brains of WT and DCIR^−/−^ mice were dissected and stored temporarily in HBSS (Sigma Aldrich, St. Louis, MO, USA) containing 15 mM HEPES (Carl Roth, Karlsruhe, Germany) and 0.5% glucose (Carl Roth, Karlsruhe, Germany). For dissociation, brains were squashed with the top end of a syringe in a 6-well plate containing a digestion cocktail (HBSS, 1 mg/ml collagenase D, 5 U/ml DNase I; Roche, Basel, Switzerland). After 10 min of incubation at 37 °C_,_ brains were gently dissociated manually. Afterwards, a 40% Percoll centrifugation (10 min, 350×*g*, 18 °C; GE Healthcare, Chicago, IL, USA) and erythrocyte lysis were performed. To check the percentage of microglia within the glial cell mixture, cells were blocked with anti-mouse CD16/32, stained with anti-mouse CD11b-PE and anti-mouse CD45-APC and fixed in 1% PFA. Flow cytometry was performed using an Attune NxT Flow Cytometer. Data analysis was conducted with FlowJo software^97^. The purity of microglia (CD11b^+^/CD45^low+^) within MEG used for co-culture experiments ranged between 40 to 60% for both WT and DCIR^−/−^ cell suspensions.

### Microglia-enriched glial cell mixture/T cell co-cultivation

Following MEG isolation, glial cells were seeded with 4 × 10^5^ cells/ml in culture medium (IMDM medium, 10% FCS, 2 mM l-glutamine, 100 U/ml penicillin 100 µg/ml streptomycin; Pan-Biotech, Aidenbach, Germany) in a 96-well U-bottom plate and stimulated with EndoGrade ovalbumin (0.3 mg/ml, LIONEX, Braunschweig, Germany) or TMEV-OVA (MOI 200) at 37 °C for 22 h. T cells were isolated from spleens of 8 to 12 week old OT-I transgenic mice using magnetic activated cell sorting (MACS, Pan T Cell Isolation Kit II mouse, Miltenyi Biotec, Bergisch Gladbach, Germany). Purified T cells were adjusted to 1 × 10^6^ cells/ml, added to the glial cells and co-cultured at 37 °C for 48 h. After incubation, supernatants were harvested and IL-2 and IFN-γ cytokine concentrations were analysed by ELISA (murine IL-2 and IFN-γ Standard ABTS ELISA Development Kit, PeproTech, Rocky Hill, NJ, USA). Co-cultured cells were blocked with anti-mouse CD16/32, stained with anti-mouse CD8a-FITC, CD62L-PE and CD69-APC and fixed in 1% PFA. Flow cytometry was performed using an Attune NxT Flow Cytometer. Data analysis was conducted with FlowJo software^97^.

### Bone marrow-derived dendritic cells/T cell co-cultivation

To generate BMDCs, bone marrow cells were isolated from femurs and tibias of DCIR^−/−^ and C57BL/6 control mice and differentiated into BMDCs by cultivation with differentiation medium (culture medium + 10% X63-GM-CSF supernatant) at 37 °C for 8 to 10 days. Following generation and differentiation, BMDCs were seeded with 2 × 10^5^ cells/ml in culture medium in a 96-well U-bottom plate and co-cultivation was performed as described above.

### Statistical analysis

Statistical analyses were performed using SPSS for Windows (version 21, SPSS Inc., IBM Corp.)^99^ applying multiple Mann–Whitney *U* tests (Supplementary Table S4) and the statistics software R (version 4.0.4)^100^ for nonparametric two-way analysis of variance (ANOVA). Moreover, statistics software R was used for applying simple and multiple regression models to study the influence of infiltrating immune cell composition, virus load and cytokine profile on hippocampal neuronal integrity. First independent parameters were preselected by single regression models. Surviving parameters were subjected to multiple regression models, which were further reduced by automatic backwards variable selection. Due to lower sample sizes at individual time points, regression models were avoided at time-specific subgroup analyses. Instead, correlation analyses using Pearson’s correlation coefficient R regarding specific analyses at time points 7 dpi and 14 dpi were performed. Graphs were designed using GraphPad Prism software (version 8, GraphPad Software Inc., San Diego, CA, USA)^101^. Statistical tests were performed with a significance level of α = 5%.

## Supplementary Information

Supplementary Information.

## Data Availability

The data presented in this study are available on request from the corresponding author.

## References

[1] Bröer S (2017). Viral mouse models of multiple sclerosis and epilepsy: Marked differences in neuropathogenesis following infection with two naturally occurring variants of Theiler's virus BeAn strain. Neurobiol. Dis..

[2] Ludlow M (2016). Neurotropic virus infections as the cause of immediate and delayed neuropathology. Acta Neuropathol..

[3] Betourne A (2018). Hippocampal expression of a virus-derived protein impairs memory in mice. Proc. Natl. Acad. Sci. USA..

[4] De Chiara G (2019). Recurrent herpes simplex virus-1 infection induces hallmarks of neurodegeneration and cognitive deficits in mice. PLoS Pathog..

[5] Vezzani A (2016). Infections, inflammation and epilepsy. Acta Neuropathol..

[6] Stewart KA, Wilcox KS, Fujinami RS, White HS (2010). Theiler's virus infection chronically alters seizure susceptibility. Epilepsia.

[7] Libbey JE (2008). Seizures following picornavirus infection. Epilepsia.

[8] Theiler M (1934). Spontaneous encephalomyelitis of mice: A new virus disease. Science.

[9] Libbey JE, Fujinami RS (2011). Neurotropic viral infections leading to epilepsy: Focus on Theiler's murine encephalomyelitis virus. Future Virol..

[10] Gerhauser I, Hansmann F, Ciurkiewicz M, Löscher W, Beineke A (2019). Facets of Theiler’s Murine encephalomyelitis virus-induced diseases: An update. Int. J. Mol. Sci..

[11] Buenz EJ, Rodriguez M, Howe CL (2006). Disrupted spatial memory is a consequence of picornavirus infection. Neurobiol. Dis..

[12] Umpierre AD (2014). Impaired cognitive ability and anxiety-like behavior following acute seizures in the Theiler's virus model of temporal lobe epilepsy. Neurobiol. Dis..

[13] Bowen JL, Olson JK (2013). IFNgamma influences type I interferon response and susceptibility to Theiler's virus-induced demyelinating disease. Viral Immunol..

[14] Drappier M (2018). A novel mechanism of RNase L inhibition: Theiler's virus L* protein prevents 2–5A from binding to RNase L. PLoS Pathog..

[15] Li L, Ulrich R, Baumgärtner W, Gerhauser I (2015). Interferon-stimulated genes-essential antiviral effectors implicated in resistance to Theiler's virus-induced demyelinating disease. J. Neuroinflamm..

[16] Libbey JE, Kennett NJ, Wilcox KS, White HS, Fujinami RS (2011). Interleukin-6, produced by resident cells of the central nervous system and infiltrating cells, contributes to the development of seizures following viral infection. J. Virol..

[17] Waltl I (2018). Microglia have a protective role in viral encephalitis-induced seizure development and hippocampal damage. Brain. Behav. Immun..

[18] Howe CL, Lafrance-Corey RG, Sundsbak RS, Lafrance SJ (2012). Inflammatory monocytes damage the hippocampus during acute picornavirus infection of the brain. J. Neuroinflamm..

[19] Howe CL (2012). Hippocampal protection in mice with an attenuated inflammatory monocyte response to acute CNS picornavirus infection. Sci. Rep..

[20] Kirkman NJ, Libbey JE, Wilcox KS, White HS, Fujinami RS (2010). Innate but not adaptive immune responses contribute to behavioral seizures following viral infection. Epilepsia.

[21] Cusick MF, Libbey JE, Patel DC, Doty DJ, Fujinami RS (2013). Infiltrating macrophages are key to the development of seizures following virus infection. J. Virol..

[22] Marzi A (2004). DC-SIGN and DC-SIGNR interact with the glycoprotein of Marburg virus and the S protein of severe acute respiratory syndrome coronavirus. J. Virol..

[23] Curtis BM, Scharnowske S, Watson AJ (1992). Sequence and expression of a membrane-associated C-type lectin that exhibits CD4-independent binding of human immunodeficiency virus envelope glycoprotein gp120. Proc. Natl. Acad. Sci. USA..

[24] Tassaneetrithep B (2003). DC-SIGN (CD209) mediates dengue virus infection of human dendritic cells. J. Exp. Med..

[25] Simmons G (2003). DC-SIGN and DC-SIGNR bind ebola glycoproteins and enhance infection of macrophages and endothelial cells. Virology.

[26] Monteiro JT, Lepenies B (2017). Myeloid C-type lectin receptors in viral recognition and antiviral immunity. Viruses.

[27] Mayer S, Raulf MK, Lepenies B (2017). C-type lectins: Their network and roles in pathogen recognition and immunity. Histochem. Cell Biol..

[28] Billadeau DD, Leibson PJ (2002). ITAMs versus ITIMs: Striking a balance during cell regulation. J. Clin. Invest..

[29] Burshtyn DN, Yang W, Yi T, Long EO (1997). A novel phosphotyrosine motif with a critical amino acid at position-2 for the SH2 domain-mediated activation of the tyrosine phosphatase SHP-1. J. Biol. Chem..

[30] Bates EE (1999). APCs express DCIR, a novel C-type lectin surface receptor containing an immunoreceptor tyrosine-based inhibitory motif. J. Immunol..

[31] Eklöw C (2008). Cellular distribution of the C-type II lectin dendritic cell immunoreceptor (DCIR) and its expression in the rheumatic joint: Identification of a subpopulation of DCIR+ T cells. Ann. Rheum. Dis..

[32] Veillette A, Latour S, Davidson D (2002). Negative regulation of immunoreceptor signaling. Annu. Rev. Immunol..

[33] Fujikado N (2008). Dcir deficiency causes development of autoimmune diseases in mice due to excess expansion of dendritic cells. Nat. Med..

[34] Hickman SE (2013). The microglial sensome revealed by direct RNA sequencing. Nat. Neurosci..

[35] Kaifu, T. & Iwakura, Y. in *C-Type Lectin Receptors in Immunity* (ed S. Yamasaki), 101–113 (Springer, 2016).

[36] Troegeler A (2017). C-type lectin receptor DCIR modulates immunity to tuberculosis by sustaining type I interferon signaling in dendritic cells. Proc. Natl. Acad. Sci. USA.

[37] Long KM (2013). Dendritic cell immunoreceptor regulates *Chikungunya* virus pathogenesis in mice. J. Virol..

[38] Maglinao M, Klopfleisch R, Seeberger PH, Lepenies B (2013). The C-type lectin receptor DCIR is crucial for the development of experimental cerebral malaria. J. Immunol..

[39] Ciurkiewicz M (2018). Cytotoxic CD8(+) T cell ablation enhances the capacity of regulatory T cells to delay viral elimination in Theiler's murine encephalomyelitis. Brain Pathol..

[40] Uhde AK (2018). Intact interleukin-10 receptor signaling protects from hippocampal damage elicited by experimental neurotropic virus infection of SJL mice. Sci. Rep..

[41] Ciurkiewicz M, Herder V, Beineke A (2020). Beneficial and detrimental effects of regulatory T cells in neurotropic virus infections. Int. J. Mol. Sci..

[42] Burrack KS, Morrison TE (2014). The role of myeloid cell activation and arginine metabolism in the pathogenesis of virus-induced diseases. Front. Immunol..

[43] Stoermer KA (2012). Genetic ablation of arginase 1 in macrophages and neutrophils enhances clearance of an arthritogenic alphavirus. J. Immunol..

[44] Oleszak EL, Chang JR, Friedman H, Katsetos CD, Platsoucas CD (2004). Theiler's virus infection: A model for multiple sclerosis. Clin. Microbiol. Rev..

[45] Chang JR, Zaczynska E, Katsetos CD, Platsoucas CD, Oleszak EL (2000). Differential expression of TGF-beta, IL-2, and other cytokines in the CNS of Theiler's murine encephalomyelitis virus-infected susceptible and resistant strains of mice. Virology.

[46] Rodriguez M (1993). Abrogation of resistance to Theiler's virus-induced demyelination in H-2b mice deficient in beta 2-microglobulin. J. Immunol..

[47] Renner DN (2016). Improved treatment efficacy of antiangiogenic therapy when combined with picornavirus vaccination in the GL261 glioma model. Neurotherapeutics.

[48] Pavelko KD (2013). The epitope integration site for vaccine antigens determines virus control while maintaining efficacy in an engineered cancer vaccine. Mol. Ther..

[49] Pavelko KD (2011). Theiler's murine encephalomyelitis virus as a vaccine candidate for immunotherapy. PLoS ONE.

[50] Maruhashi T (2015). DCIR maintains bone homeostasis by regulating IFN-gamma production in T cells. J. Immunol..

[51] Klechevsky E (2010). Cross-priming CD8+ T cells by targeting antigens to human dendritic cells through DCIR. Blood.

[52] Meyer-Wentrup F (2008). Targeting DCIR on human plasmacytoid dendritic cells results in antigen presentation and inhibits IFN-alpha production. Blood.

[53] Meyer-Wentrup F (2009). DCIR is endocytosed into human dendritic cells and inhibits TLR8-mediated cytokine production. J. Leukoc. Biol..

[54] Brinkmeyer-Langford CL (2017). Host genetic background influences diverse neurological responses to viral infection in mice. Sci. Rep..

[55] Dethlefs S, Brahic M, Larsson-Sciard EL (1997). An early, abundant cytotoxic T-lymphocyte response against Theiler's virus is critical for preventing viral persistence. J. Virol..

[56] Getts MT, Kim BS, Miller SD (2007). Differential outcome of tolerance induction in naive versus activated Theiler's virus epitope-specific CD8+ cytotoxic T cells. J. Virol..

[57] Huseby Kelcher AM (2017). Brain atrophy in picornavirus-infected FVB mice is dependent on the H-2Db class I molecule. FASEB J..

[58] Murray P, Pavelko K, Leibowitz J, Lin X, Rodriguez M (1998). CD4+ and CD8+ T cells make discrete contributions to demyelination and neurologic disease in a viral model of multiple sclerosis. J. Virol..

[59] Laidlaw BJ, Craft JE, Kaech SM (2016). The multifaceted role of CD4+ T cells in CD8+ T cell memory. Nat. Rev. Immunol..

[60] Richards MH (2011). Virus expanded regulatory T cells control disease severity in the Theiler's virus mouse model of MS. J. Autoimmun..

[61] McDole JR (2010). Rapid formation of extended processes and engagement of Theiler's virus-infected neurons by CNS-infiltrating CD8 T cells. Am. J. Pathol..

[62] Pirko I (2012). Contrasting roles for CD4 vs. CD8 T-cells in a murine model of virally induced "T1 black hole" formation. PLoS ONE.

[63] Willenbring RC (2016). Modulatory effects of perforin gene dosage on pathogen-associated blood-brain barrier (BBB) disruption. J. Neuroinflammation.

[64] Seno A (2015). Exacerbation of experimental autoimmune encephalomyelitis in mice deficient for DCIR, an inhibitory C-type lectin receptor. Exp. Anim..

[65] Hütter J (2014). Role of the C-type lectin receptors MCL and DCIR in experimental colitis. PLoS ONE.

[66] Jin YH (2010). Type I interferon signals control Theiler's virus infection site, cellular infiltration and T cell stimulation in the CNS. J. Neuroimmunol..

[67] Akdis M (2016). Interleukins (from IL-1 to IL-38), interferons, transforming growth factor beta, and TNF-alpha: Receptors, functions, and roles in diseases. J. Allergy Clin. Immunol..

[68] Rodriguez M (2009). Tumor necrosis factor alpha is reparative via TNFR2 [corrected] in the hippocampus and via TNFR1 [corrected] in the striatum after virus-induced encephalitis. Brain Pathol..

[69] Ye L (2013). IL-1β and TNF-α induce neurotoxicity through glutamate production: A potential role for neuronal glutaminase. J. Neurochem..

[70] Banchereau J, Steinman RM (1998). Dendritic cells and the control of immunity. Nature.

[71] Ueno H (2007). Dendritic cell subsets in health and disease. Immunol. Rev..

[72] Beauvillain C (2008). Neonatal and adult microglia cross-present exogenous antigens. Glia.

[73] Olson JK, Girvin AM, Miller SD (2001). Direct activation of innate and antigen-presenting functions of microglia following infection with Theiler's virus. J. Virol..

[74] Burshtyn DN, Long EO (1997). Regulation through inhibitory receptors: lessons from natural killer cells. Trends Cell Biol..

[75] Barrow AD, Trowsdale J (2006). You say ITAM and I say ITIM, let's call the whole thing off: The ambiguity of immunoreceptor signalling. Eur. J. Immunol..

[76] Klauber TCB (2017). Delivery of TLR7 agonist to monocytes and dendritic cells by DCIR targeted liposomes induces robust production of anti-cancer cytokines. Acta Biomater..

[77] du Sert NP (2020). The ARRIVE guidelines 2.0: Updated guidelines for reporting animal research. J. Cereb. Blood Flow Metab..

[78] Daniels JB, Pappenheimer AM, Richardson S (1952). Observations on encephalomyelitis of mice (DA strain). J. Exp. Med..

[79] Kumnok J (2008). Differential transcription of matrix-metalloproteinase genes in primary mouse astrocytes and microglia infected with Theiler's murine encephalomyelitis virus. J. Neurovirol..

[80] Falke D (1957). Uber die zuchtung des theiler-to-virus in der gewebekultur. Z. Hyg. Infektionskr..

[81] Lipton HL (1978). Characterization of the TO strains of Theiler's mouse encephalomyelitis viruses. Infect. Immun..

[82] Rabinowitz SG, Lipton HL (1976). Cellular immunity in chronic Theiler's virus central nervous system infection. J. Immunol..

[83] Gerhauser, I. *In vitro Expressions-Analyse von Transkriptionsfaktoren muriner Astrozyten und Mikrogliazellen nach Infektion mit dem Theiler-Enzephalomyelitis-Virus* Dr. med vet. thesis, Hannover, tierärztl. Hochschule (2009).

[84] Racine RJ (1972). Modification of seizure activity by electrical stimulation. II. Motor seizure. Electroencephalogr. Clin. Neurophysiol..

[85] Uhde AK (2016). Viral infection of the central nervous system exacerbates interleukin-10 receptor deficiency-mediated colitis in SJL mice. PLoS ONE.

[86] Gerhauser I, Alldinger S, Ulrich R, Baumgärtner W (2005). Spatio-temporal expression of immediate early genes in the central nervous system of SJL/J mice. Int. J. Dev. Neurosci..

[87] Paxinos G, Franklin KBJ (2007). The Mouse Brain in Stereotaxic Coordinates.

[88] Rattka M, Brandt C, Löscher W (2013). The intrahippocampal kainate model of temporal lobe epilepsy revisited: Epileptogenesis, behavioral and cognitive alterations, pharmacological response, and hippoccampal damage in epileptic rats. Epilepsy Res..

[89] Kummerfeld M, Meens J, Haas L, Baumgärtner W, Beineke A (2009). Generation and characterization of a polyclonal antibody for the detection of Theiler's murine encephalomyelitis virus by light and electron microscopy. J. Virol. Methods.

[90] Ulrich R (2006). MMP-12, MMP-3, and TIMP-1 are markedly upregulated in chronic demyelinating theiler murine encephalomyelitis. J. Neuropathol. Exp. Neurol..

[91] Herder V (2012). Interleukin-10 expression during the acute phase is a putative prerequisite for delayed viral elimination in a murine model for multiple sclerosis. J. Neuroimmunol..

[92] Bröer S (2016). Brain inflammation, neurodegeneration and seizure development following picornavirus infection markedly differ among virus and mouse strains and substrains. Exp. Neurol..

[93] Thornton E, Vink R, Blumbergs PC, Van Den Heuvel C (2006). Soluble amyloid precursor protein alpha reduces neuronal injury and improves functional outcome following diffuse traumatic brain injury in rats. Brain Res..

[94] SkanIT Software Version 3.2.1.4 RE. Thermo Fisher Scientific (2018). https://www.thermofisher.com/order/catalog/product/5187139#/5187139.

[95] Vandesompele J (2002). Accurate normalization of real-time quantitative RT-PCR data by geometric averaging of multiple internal control genes. Genome Biol..

[96] geNorm Version 3.4 (2002). https://genorm.cmgg.be/.

[97] FlowJo software Version 10, FloJo LLC (2020). https://www.flowjo.com/.

[98] Prajeeth CK (2014). Limited role of regulatory T cells during acute Theiler virus-induced encephalitis in resistant C57BL/6 mice. J. Neuroinflamm..

[99] SPSS for Windows, Version 21, SPSS Inc. IBM Corp. (2020). https://www.ibm.com/de-de/analytics/spss-statistics-software.

[100] The R Project for Statistical Computing Version 4.0.4 (2020). https://www.r-project.org/.

[101] GraphPad Prism Version 8, GraphPad Software Inc. (2020). https://www.graphpad.com/.

