# Supplementary material for: C-type lectin receptor DCIR contributes to hippocampal injury in acute neurotropic virus infection
Source: Sci Rep. 2021 Dec 10;11:23819. doi: 10.1038/s41598-021-03201-2 (PMC8664856; doi:10.1038/s41598-021-03201-2)
Supplement: Supplementary file 1 — Supplementary Information. [file 41598_2021_3201_MOESM1_ESM.pdf]

Supplementary file

C-type lectin receptor DCIR contributes to hippocampal injury in acute neurotropic virus infection

Melanie Stoff [1,†], Tim Ebbecke [2,3,†], Malgorzata Ciurkiewicz [1], Suvarin Pavasutthipaisit [1,2,4], Sabine Mayer-Lambertz [3], Theresa Störk [1], Kevin D. Pavelko [5], Wolfgang Baumgärtner [1,2], Klaus Jung [6], Bernd Lepenies [2,3,‡] and Andreas Beineke [1,2,‡,*]

Supplementary Methods

In vitro Theiler's murine encephalomyelitis virus Daniels strain (TMEV DA)-pulsing of bone marrow-derived dendritic cells (BMDC)

BMDCs were generated as described in "*Bone marrow-derived dendritic cells/T cell co-cultivation*". Cells were seeded with 5 x 10^5 cells/ml in culture medium (IMDM medium, 10% FCS, 2 mM L-glutamine, 100 U/ml penicillin 100 µg/ml streptomycin; Pan-Biotech, Aidenbach, Germany) in a 96-well U-bottom plate and incubated with TMEV DA (MOI 200) at 37°C for 2 h, 4 h, 6 h or 22 h. In addition, BMDC/T cell co-cultivation was performed as described using 8 x 10^5 cells/ml of BMDCs and 4 x 10^6 cells/ml of OT-I T cells. Here, as an additional stimulus, Poly(I:C) (25 µg/ml) and R837 (2,5 µg/ml) (Invivogen, San Diego, CA, USA) were used. After incubation, supernatants were harvested and IFN -β, IL-6 and TNF-α cytokine concentrations were analysed by ELISA (LumiKine Xpress mIFN-β 2.0, Invivogen, San Diego, CA, USA; mouse TNF-α or IL-6 DuoSet ELISA, R&D Systems, Minneapolis, MN, USA) or supernatants were collected to perform *in vitro* viral replication assays. TMEV-pulsed cells were either used to isolate RNA or blocked with anti-mouse CD16/32, stained with anti-mouse MHC-I-FITC, CD86-PE and CD11c-APC (or CD80-FITC, CD86-PE, CD11c-APC and MHC-I-eFluor450) and fixed in 1% PFA (Carl Roth, Karlsruhe, Germany). Flow cytometry to evaluate BMDC activation was performed using an Attune NxT Flow Cytometer (Thermo Fisher Scientific, Waltham, MA, USA). Data analysis was conducted with FlowJo software (version 10, FlowJo LLC, Ashland, OR, USA) [1].

RNA isolation, reverse transcription and in vitro Theiler's murine encephalomyelitis virus detection by quantitative polymerase chain reaction (RT-qPCR)

To isolate RNA from TMEV DA-pulsed BMDCs, cells were washed and suspended in TRI Reagent (Sigma Aldrich, St. Louis, MO, USA). Total RNA was isolated using the RNeasy extraction kit (Qiagen, Hilden, Germany) according to manufacturer's instructions. RNA purity and amount assessment as well as RNA translation into cDNA were performed as described in "*Ribonucleic acid isolation and reverse transcription*" within the main manuscript. Determination and quantification of TMEV DA RNA levels and expression of three housekeeping genes β-actin, glyceraldehyde 3-phosphate dehydrogenase (GAPDH), and hypoxanthine-guanine phosphoribosyltransferase (HPRT) was conducted as described in "*Reverse transcription - quantitative polymerase chain reaction (RT-qPCR)*" within the main manuscript. Primer details are listed in **Supplementary Table S2**.

In vitro detection of Theiler's murine encephalomyelitis virus Daniels strain (TMEV DA) replication by $TCID_{50}$

To evaluate viral titers in samples containing TMEV DA, endpoint dilution assays in BHK-21 cells were performed. One 96-well plate was used per TMEV DA supernatant sample. Briefly, all wells were filled with IMDM medium (supplemented with 2mM L-glutamine, 100 U/ml penicillin, 100 µg/ml streptomycin, and 5% FBS; Pan-Biotech, Aidenbach, Germany) prior to virus sample addition. Then TMEV DA supernatant was diluted 1:10 and added to the first column of the 96-well plate (7 technical replicates), except for the bottom well (mock control). Afterwards, virus was serially diluted along the 96-well plate, except for the last two columns (negative control). Next, a BHK-21 cells suspension was added to all wells with 100 µl/well and cells were incubated 4 – 6 days at 37°C. The 50% tissue culture infective dose ($TCID_{50}$) was obtained and focus forming units (FFU/ml) were estimated using the formula 0.69 x $TCID_{50}$, as described previously [2-5].

Viability staining of microglia-enriched glial cell mixture and bone marrow-derived dendritic cells

Following MEG isolation (cells of five animals were pooled) and BMDC generation, cells were seeded in culture medium (IMDM medium, 10% FCS, 2 mM L-glutamine, 100 U/ml penicillin 100 µg/ml streptomycin; all reagents purchased from Pan-Biotech, Aidenbach, Germany) in a 96-well U-bottom plate and stimulated with EndoGrade ovalbumin (0.3 mg/ml, LIONEX, Braunschweig, Germany), cell culture supernatant of non-infected BHK-21 cells (Mock), TMEV-OVA (MOI 200) or TMEV DA (MOI 200) at

37 °C for 22 h. After incubation, cells were blocked with anti-mouse CD16/32, MEG were stained with anti-mouse CD11b-FITC and CD45-PE-Cy7 and BMDC were stained with anti-mouse CD11c-PE. Subsequently, cells were incubated with 7-AAD viability staining solution for 5 minutes and flow cytometry was performed using an Attune NxT Flow Cytometer. As a control, MEGs and BMDCs were UV-irradiated for 10 minutes prior to the staining procedure. Data analysis was conducted with FlowJo software [1].

Supplementary Figures

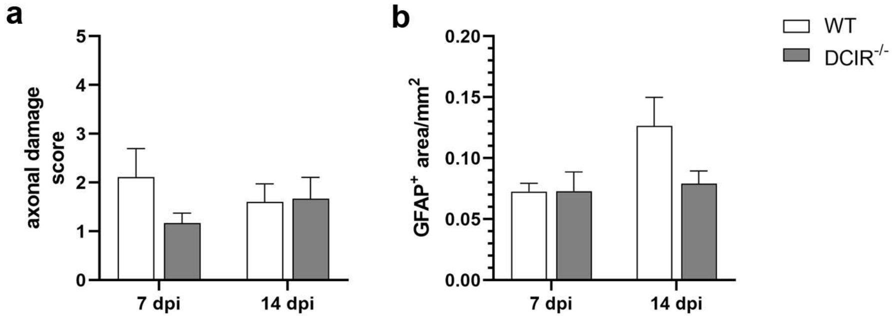

Figure S1. Immunohistochemical analysis of axonal damage and astrogliosis during the course of Theiler's murine encephalomyelitis in wild type (WT) and DCIR$^{-/-}$ mice. (**a**) Semiquantitative assessment of axonal damage within the hippocampus by beta amyloid precursor protein (β-APP)-specific immunohistochemistry. (**b**) Densitometric analysis of glial fibrillary acidic protein (GFAP) positive astrocytes within the hippocampus by immunohistochemistry. (a, b) Statistical analysis: Mann-Whitney U test, data are shown as mean with SEM. n: 7 dpi = 9 WT and 12 DCIR$^{-/-}$ mice; 14 dpi = 10 WT and 9 DCIR$^{-/-}$ mice. dpi = days post infection.

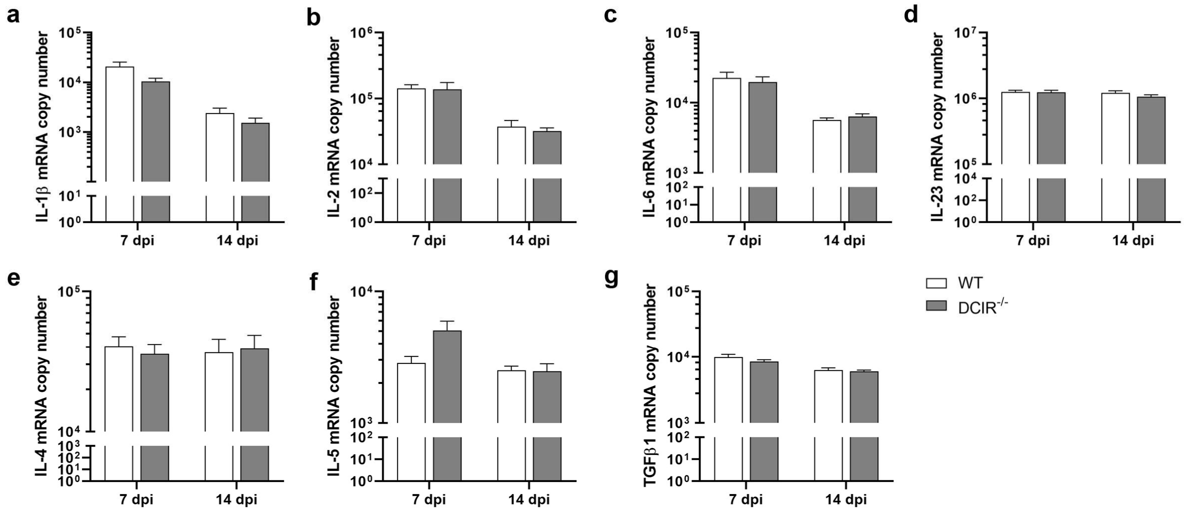

Figure S2. Additional cytokine expression data from the cerebrum of mice infected with Theiler's murine encephalomyelitis virus. Quantification of (**a**) interleukin (IL)-1β, (**b**) IL-2, (**c**) IL-6, (**d**) IL-23, (**e**) IL-4, (**f**) IL-5 and (**g**) transforming growth factor β1 (TGFβ1) mRNA in the cerebrum of wild type (WT) and DCIR$^{-/-}$ mice by reverse transcriptase quantitative polymerase chain reaction. (a-g) Statistical analysis: Mann-Whitney U test, data are shown as mean with SEM. n: 7 dpi = 9 WT and 12 DCIR$^{-/-}$ mice; 14 dpi = 10 WT and 9 DCIR$^{-/-}$ mice. dpi = days post infection.

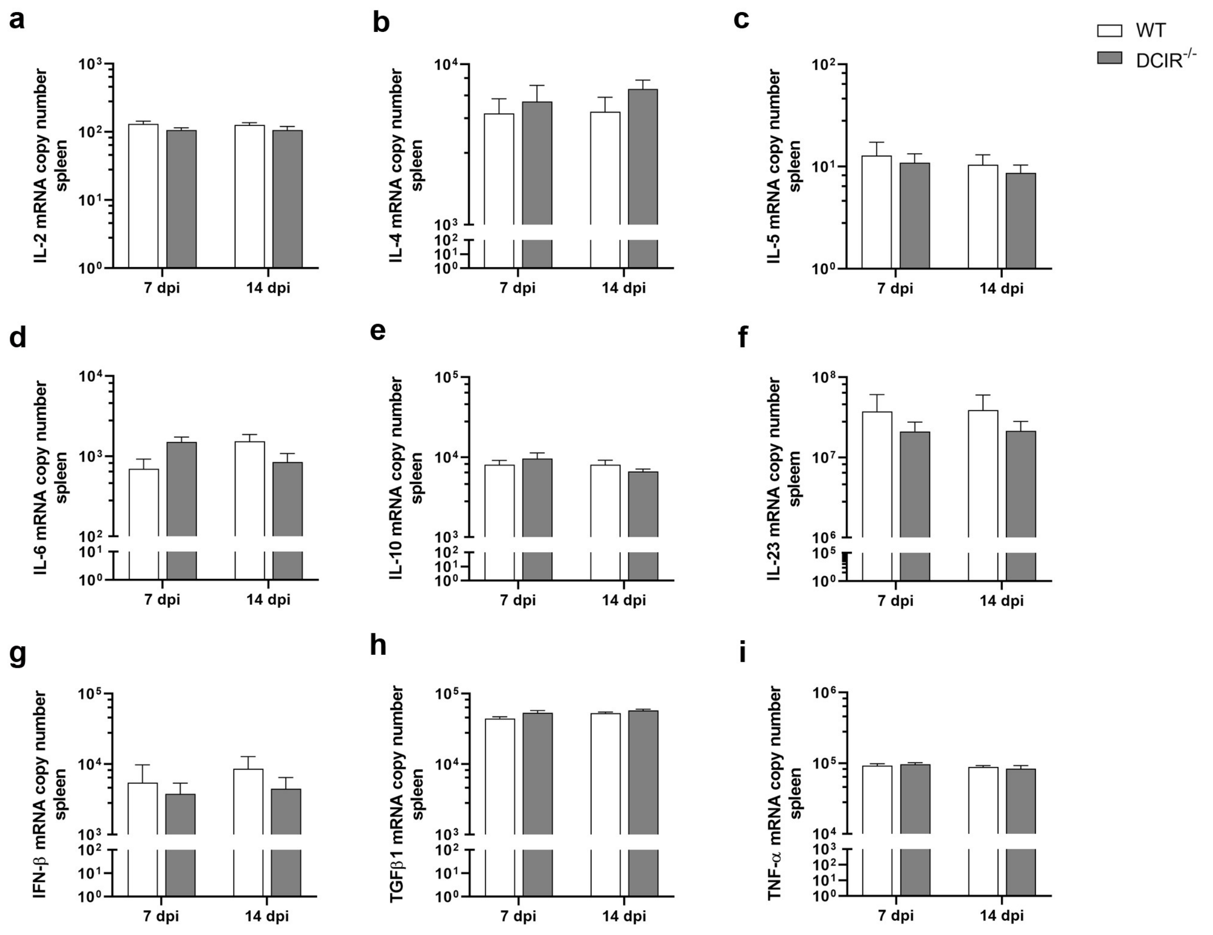

Figure S3. Splenic cytokine expression of mice infected with Theiler's murine encephalomyelitis virus. Quantification of (**a**) interleukin (IL)-2, (**b**) IL-4, (**c**) IL-5, (**d**) IL-6, (**e**) IL-10, (**f**) IL-23, (**g**) interferon (IFN)-β, (**h**) transforming growth factor β1 (TGFβ1) and (**i**) tumor necrosis factor (TNF)-α mRNA in the spleen of wild type (WT) and DCIR$^{-/-}$ mice by reverse transcriptase quantitative polymerase chain reaction. (a-i) Statistical analysis: Mann-Whitney U test, data are shown as mean with SEM. n: 7 dpi = 9 WT and 12 DCIR$^{-/-}$ mice; 14 dpi = 10 WT and 9 DCIR$^{-/-}$ mice. dpi = days post infection.

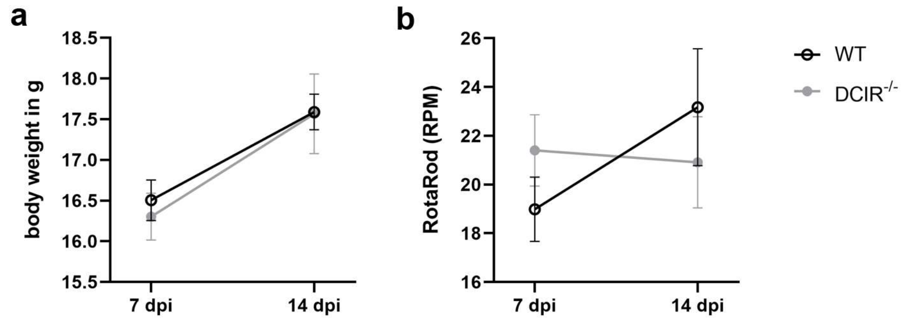

Figure S4. Body weight records (**a**) and RotaRod measurements (**b**) during the course of Theiler's murine encephalomyelitis in wild type (WT) and DCIR$^{-/-}$ mice. (**a**) At 7 and 14 dpi no significant body weight differences between the groups were detected (7 dpi: $p = 0.605$, 14 dpi: $p = 0.965$). (**b**) RotaRod performance test revealed no statistical differences between the groups during the course of TMEV infection (7 dpi: $p = 0.232$, 14 dpi: $p = 0.474$). Statistical analysis: Student's t-test, data are shown as mean with SEM. n: 7 dpi: 19 WT and 21 DCIR$^{-/-}$ mice; 14 dpi: 10 WT and 9 DCIR$^{-/-}$ mice. RPM = rounds per minute.

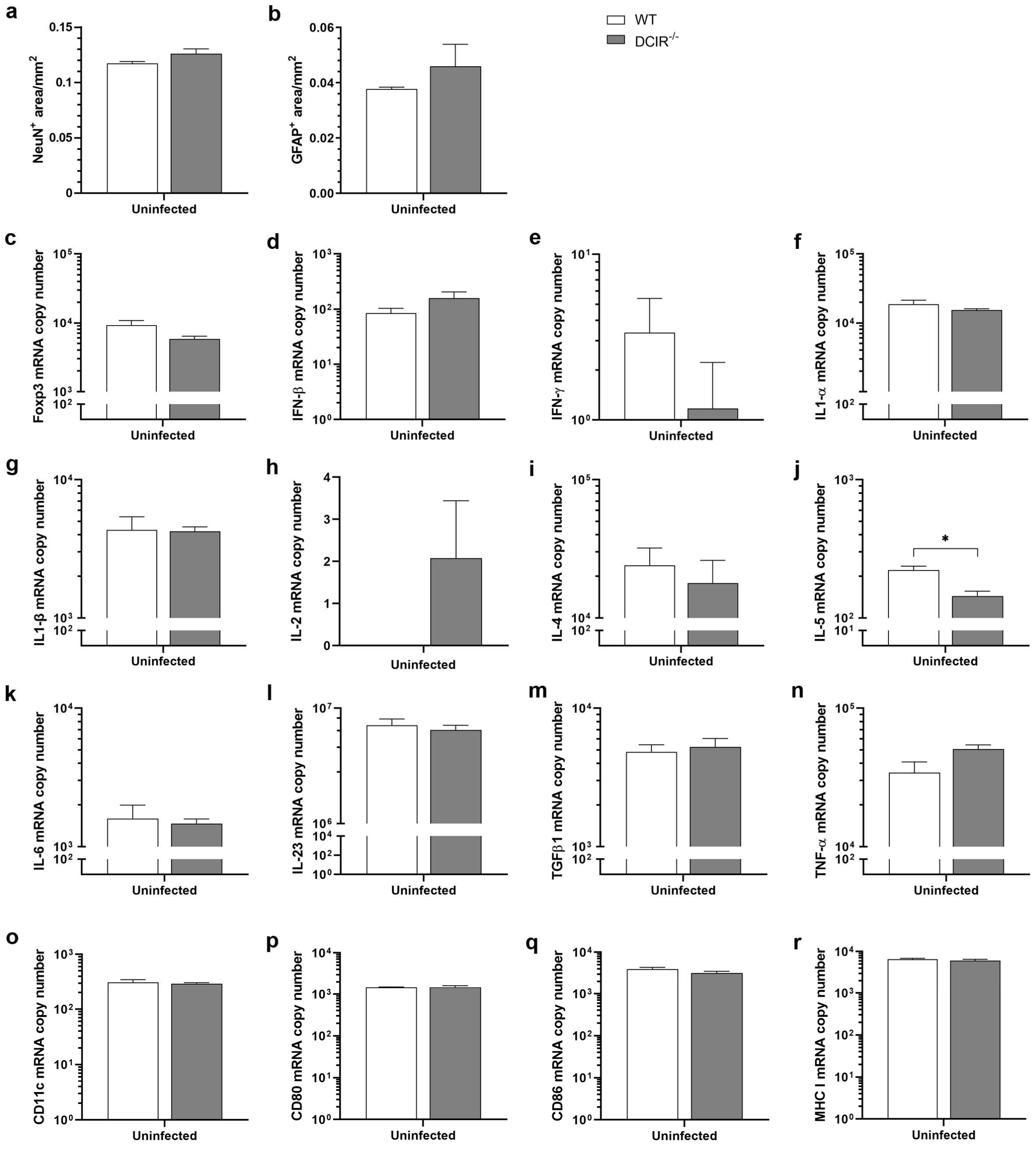

Figure S5. Immunohistochemical analyses and cytokine expression data from uninfected control mice. Densitometric analysis of (**a**) hippocampal neuronal density by NeuN-specific immunohistochemistry and (**b**) glial fibrillary acidic protein (GFAP) positive astrocytes within the hippocampus. Quantification of (**c**) forkhead box protein 3 (Foxp3), (**d**) interferon (IFN)-β, (**e**) IFN-γ, (**f**) interleukin (IL)-1α, (**g**) IL-1β, (**h**) IL-2, (**i**) IL-4, (**j**) IL-5, (**k**) IL-6, (**l**) IL-23, (**m**) transforming growth factor β1 (TGFβ1), (**n**) tumor necrosis factor (TNF)-α, (**o**) CD11c, (**p**) CD80, (**q**) CD86 and (**r**) MHC I mRNA in the cerebrum of non-infected wild type (WT) and DCIR$^{-/-}$ mice by reverse transcriptase quantitative polymerase chain reaction. IL-10 mRNA expression was not detectable by RT-qPCR, (a-r) Statistical analysis: Mann-Whitney U test (statistical differences: * = $p \leq 0.05$), data are shown as mean with SEM. n: 4 WT and 4 DCIR$^{-/-}$ mice.

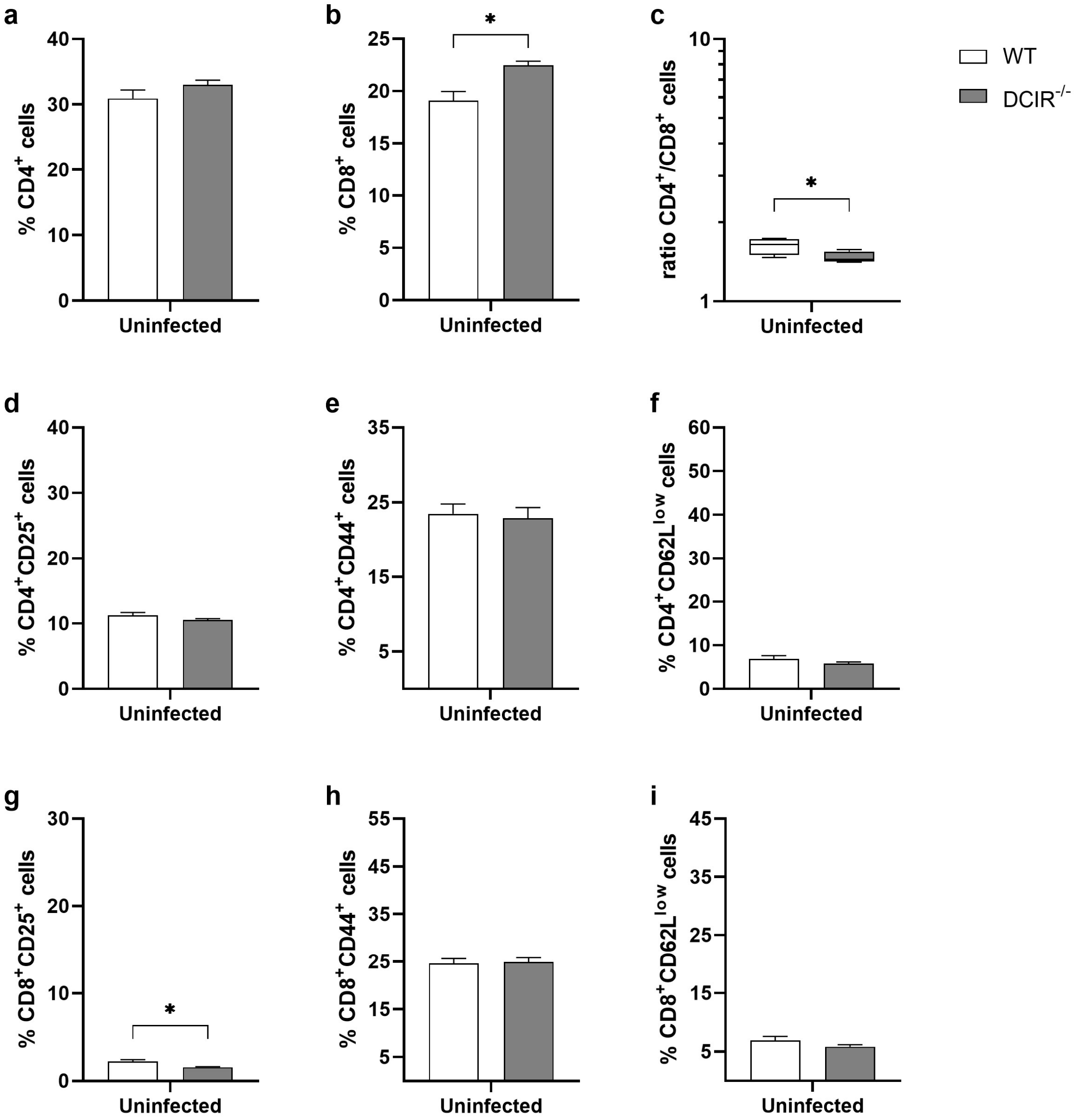

Figure S6. Flow cytometric analysis of splenic T cells in uninfected DCIR$^{-/-}$ and wild type (WT) mice. Percentages of (**a**) CD4$^+$ T cells and (**b**) CD8$^+$ T cells. (**c**) Ratio of CD4$^+$ T cells to CD8$^+$ T cells. Percentages of (**d**) CD4$^+$CD25$^+$ T cells, (**e**) CD4$^+$CD44$^+$ T cells, (**f**) CD4$^+$CD62L^{low} T cells, (**g**) CD8$^+$CD25$^+$ T cells, (**h**) CD8$^+$CD44$^+$ T cells and (**i**) CD8$^+$CD62L^{low} T cells in spleen samples. (a-i) Statistical analysis: Mann-Whitney U test (statistical differences: * = $p \leq 0.05$), (a, b, d-i) data are shown as mean with SEM and (c) box plots display median with 5-95% percentiles. n: 4 WT and 4 DCIR$^{-/-}$ mice.

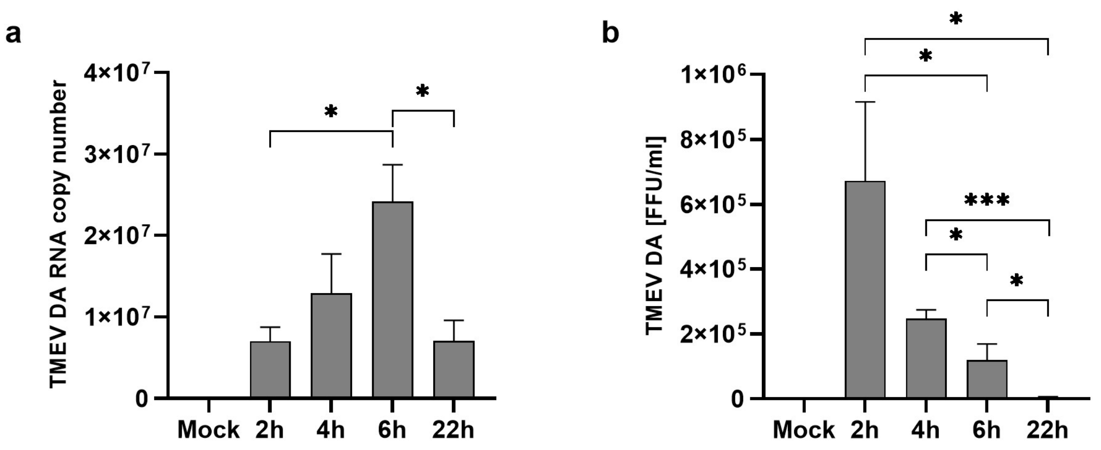

Figure S7. Viral loads in wild type (WT) bone marrow-derived dendritic cells (BMDC) challenged with Theiler's murine encephalomyelitis virus Daniel's strain (TMEV DA). (**a**) Quantification of TMEV DA (viral protein 2; VP2) RNA in WT BMDCs by reverse transcriptase quantitative polymerase chain reaction following incubation of BMDCs with TMEV DA at a MOI 200 for indicated time points. (**b**) Virus titer in the supernatant of WT BMDCs following incubation of BMDCs with TMEV DA at a MOI 200 for indicated time points analysed by $TCID_{50}$ assay. Statistical analysis: (a) Mann-Whitney U test and (b) Student's t-test (statistical differences: * = $p \leq 0.05$, ** = $p \leq 0.01$, *** = $p \leq 0.001$), data are shown as mean with SEM of n = 3.

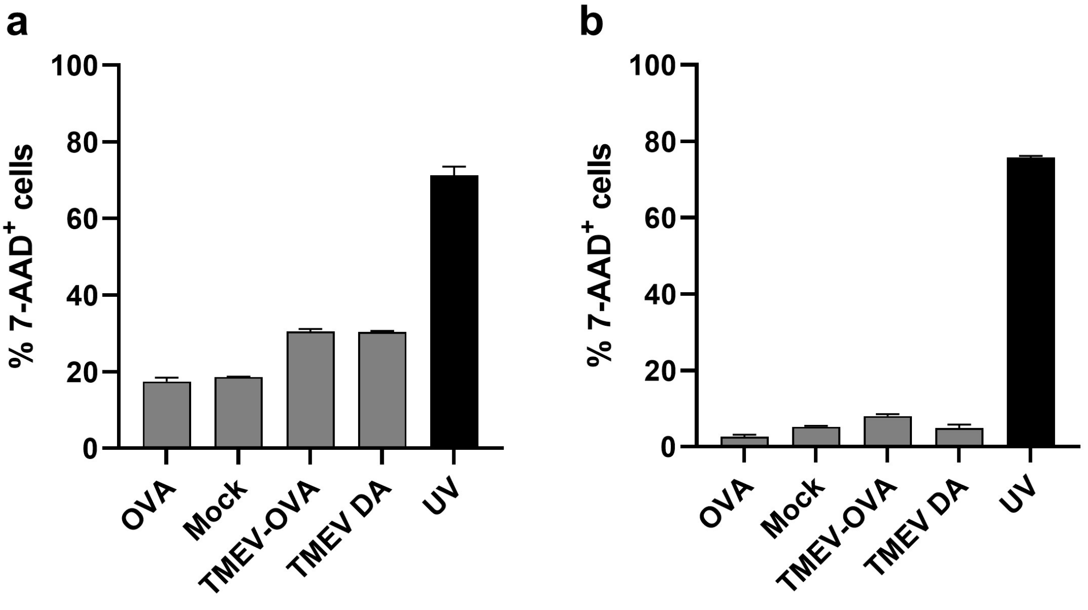

Figure S8. Flow cytometric viability analysis of wild type (WT) (**a**) microglia-enriched glial cell mixture (MEG) and (**b**) bone marrow-derived dendritic cells (BMDC) irradiated with UV-light or challenged with ovalbumin (OVA), Mock, Theiler's murine encephalomyelitis virus Daniel's strain (TMEV DA) or ovalbumin peptide-expressing TMEV (TMEV-OVA). (a, b) Percentages of $7\text{-}AAD^+$ cells following incubation of MEGs BMDCs with TMEV DA or TMEV-OVA at a MOI 200 for 22h. Statistical analysis: (**a**) Mann-Whitney U test, data are shown as mean with SEM.

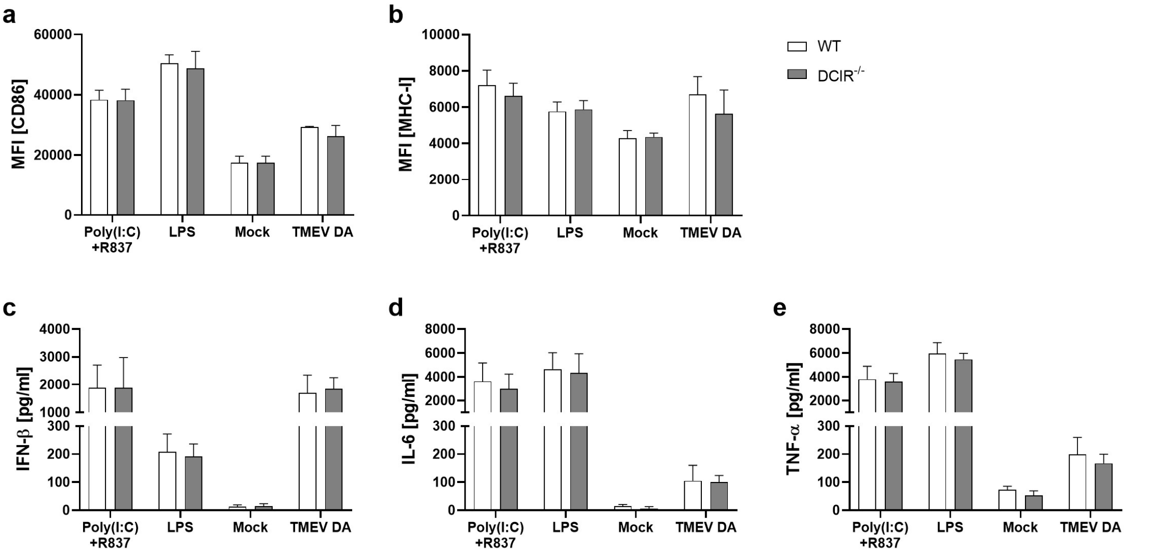

Figure S9. Flow cytometric assessment of mean fluorescence intensity (MFI) of activation markers (**a**) CD86 and (**b**) MHC-I, necessary for cross-presentation, gated on BMDCs. Analysis of the release of the cytokines (**c**) interferon (IFN)-β, (**d**) interleukin (IL)-6 and (**e**) tumor necrosis factor (TNF)-α by ELISA following BMDC stimulation. Stimulation was performed with Poly(I:C) and R837 (positive control), lipopolysaccharide (LPS, positive control), medium (Mock, negative control) or TMEV DA. (a-e) Statistical analysis: Mann-Whitney U test,data are shown as mean with SEM of n = 3.

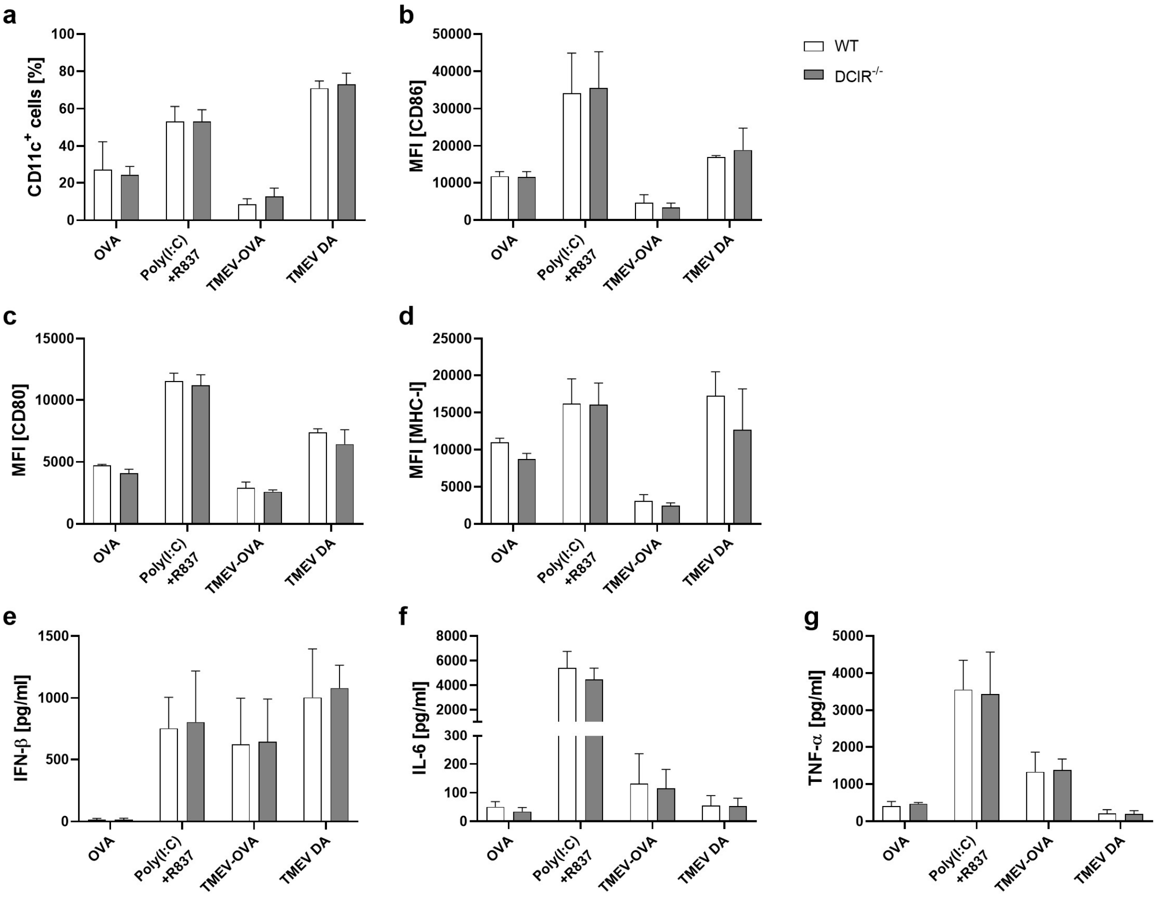

Figure S10. Flow cytometric assessment of (**a**) the percentage of CD11c⁺ cells within the whole population and mean fluorescence intensity (MFI) of activation markers (**b**) CD86, (**c**) CD80 and (**d**) MHC-I, gated on BMDCs. Analysis of the release of the cytokines (**e**) interferon (IFN)-β, (**f**) interleukin (IL)-6 and (**g**) tumor necrosis factor (TNF)-α by ELISA. All analyses were performed following a BMDC/T

cell co-cultivation using BMDCs previously challenged with ovalbumin (OVA), Poly(I:C) and R837, ovalbumin peptide-expressing TMEV (TMEV-OVA) or Theiler's murine encephalomyelitis virus Daniel's strain (TMEV DA). (**a-g**) Statistical analysis: Mann-Whitney U test, data are shown as mean with SEM of n = 3.

Supplementary Tables

Table S1. Overview of immunohistochemical staining procedures.

Antibody/ Dilution	Supplier (clone/catalogue number)	Pre-treatment	Secondary antibody	Blocking serum	Specificity
TMEV 1:2000 (pc)	Department of Pathology, University of Veterinary Medicine Hannover, Germany*	-	goat anti- rabbit	goat	TMEV capsid protein VP1
CD107b 1:200 (mc)	Bio-Rad AbD Serotec GmbH, Puchheim, Germany (clone M3/84)	20 min citrate buffer/MW	rabbit anti-rat	rabbit	activated macrophages/ microglia
arginase 1 1:200 (pc)	Thermo Fisher Scientific, Invitrogen, Waltham, MA, USA, (PA5-29645)	20 min citrate buffer/MW	goat anti-rabbit	goat	M2-type macrophages/ microglia
CD3 1:500 (pc)	Dako/Agilent Technologies, Santa Clara, CA, USA (A0452)	20 min citrate buffer/MW	goat anti-rabbit	goat	T cells
CD45R 1:1000 (biotinylated mc)	BD Bioscience, Heidelberg, Germany, (clone RA3-6B2)	20 min citrate buffer/MW	-	-	B cells
Foxp3 1:20 (mc)	Thermo Fisher Scientific, Invitrogen, eBioscience™, Waltham, MA, USA (clone FJK-16s)	20 min citrate buffer/MW	rabbit anti-rat	rabbit	regulatory T cells
NeuN 1:1600 (mc)	Merck Millipore, Darmstadt, Germany (clone A60)	20 min citrate buffer/MW	goat anti-mouse	goat	neurons
β-APP 1:2000 (mc)	Merck Millipore, Darmstadt, Germany (clone 22C11)	20 min citrate buffer/MW	goat anti-mouse	goat	damaged axons
GFAP 1:1000 (pc)	Dako/Agilent Technologies, Santa Clara, CA, USA (Z0334)	-	goat anti rabbit	goat	astrocytes
CD4 1:2000 (mc)	BD Bioscience, Heidelberg, Germany (clone RM4-5)	-	rabbit anti-rat	rabbit	$CD4^+$ T cells
CD8 1:1000 (mc)	BD Bioscience, Heidelberg, Germany, (clone 53-5.8)	-	rabbit anti-rat	rabbit	$CD8^+$ T cells
granzyme B 1:400 (pc)	Abcam, Cambridge, UK, pc	20 min citrate buffer/MW	goat anti-rabbit	goat	granzyme B^+ NK and T cells

β-APP = beta-amyloid precursor protein; Foxp3 = forkhead box P3; GFAP = glial fibrillary acidic protein; mc = monoclonal; MW = microwave oven; NeuN = neuronal nuclei; pc = polyclonal; TMEV = Theiler's murine encephalomyelitis virus; NK cells = natural killer cells; * Kummerfeld et al. [6]

Table S2. Summary of genes and primer pair sequences used for reverse transcriptase quantitative polymerase chain reaction.

Gene	Accession No.	mRNA position	Primer direction	Primer sequence from 5' → 3'	Size (bp)	Reference
HPRT	NM_013556.2	646 - 665	forward	GGA CCT CTC GAA GTG TTG GA	188	[7]
		833 - 814	reverse	TCG TAT TTG CAG ATT CAA CT		
GAPDH	NM_001289726.1	351 - 370	forward	GAG GCC GGT GCT GAG TAT GT	288	[7]
		638 - 619	reverse	GGT GGC AGT GAT GGC ATG GA		
β-actin	NM_007393.5	698 - 717	forward	GGC TAC AGC TTC ACC ACC AC	233	[7]
		930 - 911	reverse	ATG CCA CAG GAT TCC ATA CC		
CD11c	NM_021334.3	2396 - 2417	forward	TGCCAGGATGACCTTAGTGTCG	135	[8]
		2530 - 2509	reverse	CAGAGTGACTGTGGTTCCGTAG		
CD80	NM_001359898.1	287 - 309	forward	CCTCAAGTTTCCATGTCCAAGGC	140	[8]
		426 - 405	reverse	GAGGAGAGTTGTAACGGCAAGG		
CD86	NM_019388.3	837 - 860	forward	ACGTATTGGAAGGAGATTACAGCT	147	[8]
		983 - 962	reverse	TCTGTCAGCGTTACTATCCCGC		
IL-1α	NM_010554.4	294 - 313	forward	AAG CAA CGG GAA GAT TCT GA	179	[9]
		472 - 453	reverse	TGA CAA ACT TCT GCC TGA CG		
IL-1β	NM_008361.4	311 - 330	forward	AGC TAC CTG TGT CTT TCC CG	150	[10]
		460 - 439	reverse	AGT GCA GTT GTC TAA TGG GAA C		
IL-2	NM_008366.3	227 - 247	forward	GCA GGA TGG AGA ATT ACA GGA	183	[9]
		409 - 388	reverse	TGA AAT TCT CAG CAT CTT CCA A		
IL-4	NM_021283.2	224 - 245	forward	CCT CAC AGC AAC GAA GAA CAC C	156	[9]
		379 - 358	reverse	CAT CGA AAA GCC CGA AAG AGT C		
IL-5	NM_010558.1	104 - 123	forward	ATG GAG ATT CCC ATG AGC AC	180	[11]
		283 - 264	reverse	CCC ACG GAC AGT TTG ATT CT		
IL-6	NM_031168.2	250 - 269	forward	GTT CTC TGG GAA ATC GTG GA	176	[11]
		425 - 404	reverse	CCA GAG GAA ATT TTC AAT AGG C		
IL-10	NM_010548.2	307 - 326	forward	CCA AGC CTT ATC GGA AAT GA	162	[9]
		468 - 449	reverse	TTT TCA CAG GGG AGA AAT CG		
IL-23	NM_031252.2	517 - 538	forward	CCT CCA GCC AGA GGA TCA CCC C	145	[12]
		661 - 642	reverse	GTG GGC AAA GAC CCG GGC AG		
Foxp3	NM_054039.2	801 - 821	forward	CCC AGG AAA GAC AGC AAC CTT	89	[12]
		889 - 869	reverse	TTC TCA CAA CCA GGC CAC TTG		
IFN-β	NM_010510.1	154 - 178	forward	TGA ATG GAA AGA TCA ACC TCA CCT A	76	[13]
		229 - 208	reverse	CTC TTC TGC ATC TTC TCC GTC A		
IFN-γ	NM_008337.4	176 - 195	forward	CAC GGC ACA GTC ATT GAA AG	144	[14]
		319 - 300	reverse	AAT CTG GCT CTG CAG GAT TT		
MHC I [H2-K]	NM_001001892.2	344 – 365	forward	GGCAATGAGCAGAGTTTCCGAG	106	[8]
		449-428	reverse	CCACTTCACAGCCAGAGATCAC		
TGFβ1	NM_011577.2	1719 - 1738	forward	TTG CTT CAG CTC CAC AGA GA	183	[14]
		1901 - 1882	reverse	TGG TTG TAG AGG GCA AGG AC		
TMEV DA VP2	M.20301.1	1596 - 1615	forward	TGG TCG ACT CTG TGG TTA CG	238	[15]
		1833 - 1814	reverse	GCC GGT CTT GCA AAG ATA GT		
TNF-α	NM_013693.3	268 - 287	forward	GCC TCT TCT CAT TCC TGC TT	203	[14]
		470 - 451	reverse	CAC TTG GTG GTT TGC TAC GA		

Accession No. = GenBank sequence accession number and version (National Center for Biotechnology Information, U.S. National Library of Medicine, Bethesda MD, USA), bp: base pairs, GAPDH = glyceraldehyde 3-phosphate dehydrogenase, HPRT = hypoxanthine-guanine

phosphoribosyltransferase, Foxp3 = Forkhead box p3, IFN = interferon, IL = interleukin, TNF-α = tumor necrosis factor α, TGFβ1 = transforming growth factor β1

Table S3. Overview of monoclonal antibodies used for flow cytometry.

Antibody and conjugate	Supplier (clone)	Dilution	Specificity
CD4-FITC	BD Biosciences, BD Pharmingen^{TM}, Heidelberg, Germany (clone GK1.5)	1:100	$CD4^+$ helper T cells
CD4-FITC	Thermo Fisher Scientific, Invitrogen, eBioscience^{TM}, Waltham, MA, USA (clone RM4-5)	1:100	$CD4^+$ helper T cells
CD4-PE	BD Biosciences, BD Pharmingen^{TM}, Heidelberg, Germany (clone GK1.5)	1:100	$CD4^+$ helper T cells
CD4-PerCP Cy5.5	Thermo Fisher Scientific, Invitrogen, eBioscience^{TM}, Waltham, MA, USA (clone RM4-5)	1:200	$CD4^+$ helper T cells
CD8a-FITC	Thermo Fisher Scientific, Invitrogen, eBioscience^{TM}, Waltham, MA, USA (clone 53-6.7)	1:100	$CD8^+$ cytotoxic T cells
CD8a-APC	BD Biosciences, BD Pharmingen^{TM}, Heidelberg, Germany, clone 53-6.7)	1:200	$CD8^+$ cytotoxic T cells
CD8a-PE	BD Biosciences, BD Pharmingen^{TM}, Heidelberg, Germany (clone 53-6.7)	1:200	$CD8^+$ cytotoxic T cells
CD11b-FITC	BD Biosciences, BD Pharmingen^{TM}, Heidelberg, Germany (clone M1/70)	1:100	Macrophages/microglia
CD11b-PE	Thermo Fisher Scientific, Invitrogen, eBioscience^{TM}, Waltham, MA, USA (clone M1/70)	1:100	Macrophages/microglia
CD11c-PE	Thermo Fisher Scientific, Invitrogen, eBioscience^{TM}, Waltham, MA, USA (clone N418)	1:100	Dendritic cells
CD11c-APC	Elabscience, Houston, TX, USA (clone N418)	1:200	Dendritic cells
CD19-FITC	Thermo Fisher Scientific, Invitrogen, eBioscience^{TM}, Waltham, MA, USA (clone eBio 1D3)	1:100	B cells
CD25-FITC	BD Biosciences, BD Pharmingen^{TM}, Heidelberg, Germany (clone 3C7)	1:100	Activated T cells, regulatory T cells
CD44-APC	BD Biosciences, BD Pharmingen^{TM}, Heidelberg, Germany (clone IM7)	1:200	Activated T cells, memory T cells
CD45-APC	Thermo Fisher Scientific, Invitrogen, eBioscience^{TM}, Waltham, MA, USA (clone 30-F11)	1:100	Lymphocytes/microglia
CD45-PE-Cy7	Thermo Fisher Scientific, Invitrogen, eBioscience^{TM}, Waltham, MA, USA (clone 30-F11)	1:200	Lymphocytes/microglia
CD62L-PE	BD Biosciences, BD Pharmingen^{TM}, Heidelberg, Germany (clone MEL-14)	1:200	T cell activation marker, downregulation upon T cell activation, L-selectin
CD62L-PE-Cy7	Thermo Fisher Scientific, Invitrogen, eBioscience^{TM}, Waltham, MA, USA (clone MEL-14)	1:200	T cell activation marker, downregulation upon T cell activation, L-selectin
CD69-APC	Thermo Fisher Scientific, Invitrogen, eBioscience^{TM}, Waltham, MA, USA (clone H1.2F3)	1:200	T cell activation marker, upregulation upon T cell activation
CD80-FITC	Thermo Fisher Scientific, Invitrogen, eBioscience^{TM}, Waltham, MA, USA (clone 16-10A1)	1:200	DC activation marker, upregulation upon DC activation
CD86-PE	Thermo Fisher Scientific, Invitrogen, eBioscience^{TM}, Waltham, MA, USA (clone GL1)	1:200	DC activation marker, upregulation upon DC activation
MHC-I-FITC	Thermo Fisher Scientific, Invitrogen, eBioscience^{TM}, Waltham, MA, USA (clone AF6-88.5.5.3)	1:100	Surface marker involved in cross-presentation
MHC-I-eFluor450	Thermo Fisher Scientific, Invitrogen, eBioscience^{TM}, Waltham, MA, USA (clone AF6-88.5.5.3)	1:200	Surface marker involved in cross-presentation
7-AAD	Thermo Fisher Scientific, Invitrogen, eBioscience^{TM}, Waltham, MA, USA	1:80	Viability staining solution

APC = allophycocyanin, Cy = cyanine dye, FITC = fluorescein isothiocyanate, PE = phycoerythrin, PerCP = peridinin chlorophyll protein complex

Table S4. Results of statistical analyses of in vivo experiments (*p* values).

Analysis	Marker	*p* value	
		**7 dpi**	**14 dpi**
Histology	HE (score)	0.121	**0.005****↓
Immunohistochemistry	TMEV (cells/mm^2)	0.120	**0.030***↓
	TMEV$^+$ area/mm^2	0.225	**0.005****↓
	NeuN (score)	0.098	**0.016***↓
	NeuN$^+$ area/mm^2	0.136	**0.002****↑
	CD107b$^+$ area/mm^2	0.477	0.102
	arginase 1 (cells/mm^2)	0.075	**0.005****↓
	CD3$^+$ area/mm^2	0.831	**0.007****↓
	CD45R (cells/mm^2)	0.434	**0.003***↓
	Foxp3 (cells/mm^2)	0.320	**0.018***↓
	granzyme B (cells/mm^2)	0.887	**0.05***↓
	CD4 (cells/mm^2)	0.776	**0.002****↓
	CD8 (cells/mm^2)	0.320	**0.011***↓
	ratio CD4$^+$/CD8$^+$ cells	0.177	**0.00045****↓
	GFAP (% of positive area)	0.722	0.086
	β-APP (score)	0.196	0.862
RT-qPCR CNS	CD11c	0.111	**0.017***↓
	CD80	**0.049***↓	0.065
	CD86	**0.049***↓	**0.001****↓
	IFN-β	**0.009****↓	**0.034***↓
	IFN-γ	0.356	**0.027***↓
	IL-1α	**0.047***↓	**0.018***↓
	IL-1β	0.088	0.221
	IL-2	0.569	0.744
	IL-4	0.522	0.934
	IL-5	0.102	0.624
	IL-6	0.619	0.624
	IL-10	0.394	**0.034***↓
	MHC I	0.193	**0.003****↓
	IL-23	0.670	0.165
	Foxp3	0.256	**0.009****↓
	TGFβ1	0.394	0.462
	TMEV DA	**0.047***↓	0.870
	TNF-α	**0.039***↓	**0.022***↓
RT-qPCR spleen	CD11c	0.277	**0.035***↓
	CD80	**0.034***↓	0.356
	CD86	0.554	0.780
	IFN-β	0.917	0.905
	IFN-γ	**0.049***↑	0.315
	IL-1α	0.972	**0.006****↓
	IL-1β	0.219	**0.001****↓
	IL-2	0.095	0.182
	IL-4	0.702	0.182
	IL-5	0.887	0.594
	IL-6	0.082	0.095

	IL-10	0.808	0.661
	IL-23	0.508	0.905
	MHC I	**0.049***↓	0.211
	TGFβ1	0.136	0.243
	TNF-α	1.0	0.497
Flow cytometry of spleen samples	$CD4^+$	0.834	**0.028***↓
	$CD4^+CD62L^{low}$	**0.016***↑	**0.047***↑
	$CD4^+CD25^+$	0.175	0.117
	$CD4^+CD44^+$	**0.047***↑	0.175
	$CD8^+$	**0.016***↑	0.602
	$CD8^+CD62L^{low}$	0.142	0.347
	$CD8^+CD25^+$	0.347	0.917
	$CD8^+CD44^+$	**0.009****↑	**0.016***↑
	ratio $CD4^+/CD8^+$ cells	**0.009****↓	**0.028***↓

Statistical analysis: Mann-Whitney U test; bold values indicate statistical differences: * = $p \leq 0.05$, ** = $p \leq 0.01$; ↑↓ = significant increase (↑) or decrease (↓) in $DCIR^{-/-}$ group vs wild type group. dpi = days post infection

Table S5. Results of regression analyses with neuronal integrity ($NeuN^+$ area/mm^2) as response variable.

**Explanatory variable**	**Simple regression**	**Multiple regression**
	***p* value**	***p* value**
TMEV (cells/mm^2)	**0.0010**	**0.0140**
$CD107b^+$ area/mm^2	**0.0009**	0.1624
$CD3^+$ area/mm^2	**0.0273**	0.6796
CD45R (cells/mm^2)	**0.0012**	0.5789
Foxp3 (cells/mm^2)	**0.0021**	0.2325
arginase 1 (cells/mm^2)	**< 0.0001**	0.1388
GFAP (% positive area)	**0.0144**	0.6678
granzyme B (cells/mm^2)	**0.0267**	0.8476
CD4 (cells/mm^2)	0.7876	-
CD8 (cells/mm^2)	0.8735	-
IL-1α	0.6811	-
IL-6	0.6058	-
IFN-γ	0.9709	-
TNF-α	0.1883	-
IL-2	0.0994	-
IL-5	0.2104	-
TGFβ1	0.1941	-
IL-10	0.2646	-
Foxp3	0.1333	-
IL-23	0.4956	-
IL-4	0.2222	-
IL-1β	0.0770	-
IFN-β	**0.0262**	0.8160
group	**0.0003**	0.1396
time	0.8210	-

Table S6. Simple regression analyses between neuronal integrity, viral load and immune parameters in the brain.

correlated parameters		neuronal integrity ($NeuN^+$ area/mm^2)	
		p-value	R
**TMEV (cells/mm²)**	7 dpi	**0.0004****	-0.6995
	14 dpi	0.0585	-0.4415
**$CD107b^+$ area/mm^2**	7 dpi	**0.0210***	-0.5001
	14 dpi	**0.0216***	-0.5228
**arginase 1 (cells/mm^2)**	7 dpi	**0.0080****	-0.5624
	14 dpi	**0.0016****	-0.6716
**$CD3^+$ area/mm^2**	7 dpi	0.1227	-0.3475
	14 dpi	0.0771	-0.4152
**CD45R (cells/mm^2)**	7 dpi	**0.0207***	-0.5009
	14 dpi	**0.0317***	-0.4938
**Foxp3 (cells/mm^2)**	7 dpi	0.1488	-0.3263
	14 dpi	**0.0032****	-0.6388
**granzyme B (cells/mm^2)**	7 dpi	0.1604	-0.3178
	14 dpi	0.0944	-0.3948
**CD4 (cells/mm^2)**	7 dpi	0.6077	0.1189
	14 dpi	0.0583	-0.4417
**CD8 (cells/mm^2)**	7 dpi	0.5679	0.1322
	14 dpi	0.0849	-0.4056
**GFAP (% positive area)**	7 dpi	0.6636	-0.1009
	14 dpi	**0.0027****	-0.6474
**IFN-β**	7 dpi	**0.0001****	-0.3048
	14 dpi	0.5400	-0.1462
**IFN-γ**	7 dpi	0.8108	0.0857
	14 dpi	**0.0461***	-0.2281
**IL-1α**	7 dpi	0.6035	0.0762
	14 dpi	0.3394	-0.1345
**IL-1β**	7 dpi	**0.0286***	-0.1714
	14 dpi	0.1269	-0.2164
**IL-2**	7 dpi	**0.0371***	0.3214
	14 dpi	0.2586	-0.0877
**IL-4**	7 dpi	0.9935	-0.0144
	14 dpi	0.1111	0.2230
**IL-5**	7 dpi	0.1888	0.0857
	14 dpi	0.8064	-0.0760
**IL-6**	7 dpi	0.7505	0.0000
	14 dpi	0.1910	0.1111
**IL-10**	7 dpi	0.2657	-0.1238
	14 dpi	0.3362	-0.2047
**IL-23**	7 dpi	0.2259	-0.2095
	14 dpi	0.8002	-0.0877
**Foxp3**	7 dpi	0.5298	-0.0667
	14 dpi	0.0713	-0.2749
**TGFβ1**	7 dpi	0.2240	-0.0762
	14 dpi	0.1469	-0.3333
**TNF-α**	7 dpi	0.1039	-0.0857
	14 dpi	0.0821	-0.1930

Statistical analysis: Pearson's correlation coefficient R, bold values indicate significant correlations: * = $p \leq 0.05$, ** = $p \leq 0.01$

References

1 FlowJo software Version 10, FloJo LLC (2020). [online] https://www.flowjo.com/.
2 Reed, L. J. & Muench, H. A simple method of estimating fifty per cent endpoints. *Am. J. Epidemiol.* **27**, 493-497 (1938).
3 LaBarre, D. D. & Lowy, R. J. Improvements in methods for calculating virus titer estimates from TCID50 and plaque assays. *J. Virol. Methods* **96**, 107-126 (2001).
4 Pourianfar, H. R., Javadi, A. & Grollo, L. A colorimetric-based accurate method for the determination of enterovirus 71 titer. *Indian J. Virol.* **23**, 303-310 (2012).
5 Monteiro, J. T. *et al.* The CARD9-Associated C-Type Lectin, Mincle, Recognizes La Crosse Virus (LACV) but Plays a Limited Role in Early Antiviral Responses against LACV. *Viruses* **11**, doi:10.3390/v11030303 (2019).
6 Kummerfeld, M., Meens, J., Haas, L., Baumgärtner, W. & Beineke, A. Generation and characterization of a polyclonal antibody for the detection of Theiler's murine encephalomyelitis virus by light and electron microscopy. *J. Virol. Methods* **160**, 185-188, doi:10.1016/j.jviromet.2009.04.030 (2009).
7 Gerhauser, I., Alldinger, S., Ulrich, R. & Baumgärtner, W. Spatio-temporal expression of immediate early genes in the central nervous system of SJL/J mice. *Int. J. Dev. Neurosci.* **23**, 637-649, doi:10.1016/j.ijdevneu.2005.06.004 (2005).
8 Ye, J. *et al.* Primer-BLAST: a tool to design target-specific primers for polymerase chain reaction. *BMC Bioinformatics* **13**, 134, doi:10.1186/1471-2105-13-134 (2012).
9 Herder, V. *et al.* Cuprizone inhibits demyelinating leukomyelitis by reducing immune responses without virus exacerbation in an infectious model of multiple sclerosis. *J. Neuroimmunol.* **244**, 84-93, doi:10.1016/j.jneuroim.2012.01.010 (2012).
10 Koressaar, T. & Remm, M. Enhancements and modifications of primer design program Primer3. *Bioinformatics* **23**, 1289-1291, doi:10.1093/bioinformatics/btm091 (2007).
11 Uhde, A. K. *et al.* Viral Infection of the Central Nervous System Exacerbates Interleukin-10 Receptor Deficiency-Mediated Colitis in SJL Mice. *PLoS One* **11**, e0161883, doi:10.1371/journal.pone.0161883 (2016).
12 Ciurkiewicz, M. *et al.* Cytotoxic CD8(+) T cell ablation enhances the capacity of regulatory T cells to delay viral elimination in Theiler's murine encephalomyelitis. *Brain Pathol.* **28**, 349-368, doi:10.1111/bpa.12518 (2018).
13 Li, L., Ulrich, R., Baumgärtner, W. & Gerhauser, I. Interferon-stimulated genes-essential antiviral effectors implicated in resistance to Theiler's virus-induced demyelinating disease. *J. Neuroinflammation* **12**, 242, doi:10.1186/s12974-015-0462-x (2015).
14 Herder, V. *et al.* Lack of cuprizone-induced demyelination in the murine spinal cord despite oligodendroglial alterations substantiates the concept of site-specific susceptibilities of the central nervous system. *Neuropathol. Appl. Neurobiol.* **37**, 676-684, doi:10.1111/j.1365-2990.2011.01168.x (2011).
15 Zoecklein, L. J. *et al.* Direct comparison of demyelinating disease induced by the Daniel's strain and BeAn strain of Theiler's murine encephalomyelitis virus. *Brain Pathol.* **13**, 291-308, doi:10.1111/j.1750-3639.2003.tb00029.x (2003).
